# Supplementary figures and images for: Modeling treatment and temperature effects on dengue transmission at the division level in Bangladesh
Source: PLoS One. 2026 May 15;21(5):e0348077. doi: 10.1371/journal.pone.0348077 (PMC13178928; doi:10.1371/journal.pone.0348077)

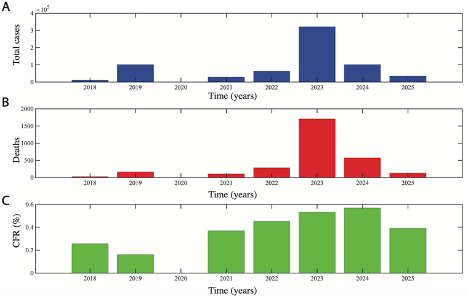

Supplement: S1 Fig — (TIF) [file pone.0348077.s001.tif]

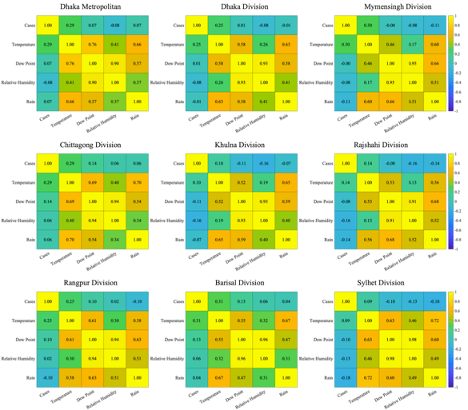

Supplement: S2 Fig — (TIF) [file pone.0348077.s002.tif]
