# Supplementary material for: Modeling treatment and temperature effects on dengue transmission at the division level in Bangladesh
Source: PLoS One. 2026 May 15;21(5):e0348077. doi: 10.1371/journal.pone.0348077 (PMC13178928; doi:10.1371/journal.pone.0348077)
Supplement: S1 Table — (PDF) [file pone.0348077.s003.pdf]

Table S1: Administrative division populations of Bangladesh, 2022 census results.

#	**Division Name**	**Population (as of 2022)**	**Reference**
**1.**	Dhaka Metropolitan Area (Greater Dhaka, not a division)	21,551,232	2022 census data [1]
**2.**	Dhaka Division	44,215,107	2022 census data [1]
**3.**	Mymensingh Division	12,225,498	2022 census data [1]
**4.**	Chittagong Division	33,202,326	2022 census data [1]
**5.**	Khulna Division	17,416,645	2022 census data [1]
**6.**	Rajshahi Division	20,353,119	2022 census data [1]
**7.**	Rangpur Divison	17,610,956	2022 census data [1]
**8.**	Barisal Division	9,100,102	2022 census data [1]
**9.**	Sylhet Division	11,034,863	2022 census data [1]
**10.**	Country total	165,158,616	(official 2022 count)

As shown in **Table S1,** the 2022 population and housing census [1] reported a national population of 165.16 million, with the Dhaka Metropolitan area alone comprising 21.55 million people. Estimated data for 2024 can be found in [2].

References

[1] Bangladesh Bureau of Statistics (2023) *Population and Housing Census 2022: Preliminary Results*. Dhaka: BBS.

[2] Worldometer (2025) Bangladesh population. Available from: Worldometer.
