# Supplementary material for: Modeling treatment and temperature effects on dengue transmission at the division level in Bangladesh
Source: PLoS One. 2026 May 15;21(5):e0348077. doi: 10.1371/journal.pone.0348077 (PMC13178928; doi:10.1371/journal.pone.0348077)
Supplement: S2 Table — (PDF) [file pone.0348077.s004.pdf]

**Table S2: Reported basic reproduction numbers ($R_0$) cross different countries**

| Country Name | Reproduction Number ($R_0$) | Sources |
|---|---|---|
| **Brazil** | 6.6 | [1] |
| **Colombia** | 1 to 9 | [2] |
| **India (Ahmedabad)** | 1.292 in 2007 to a maximum of 1.753 in 2005.<br>1.54 from 2005-2012 | [3] |
| **Indonesia** | 26.47609 | [4] |
| **Singapore** | Between 0.45 and 1.82, with a median value of 1.01 across 2010 to 2020 | [5] |
| | Between 0.54 and 2.91, with a median value of 1.02. | |
| **Cape Verde** | 2.396 | [6] |

This table summarizes reported basic reproduction numbers ($R_0$ ) for mosquito-borne diseases across different countries. The values vary significantly, with Indonesia reporting the highest $R_0 = 26.48$ while countries such as Singapore and Colombia display a broader range. These variations reflect differences in geography, climate, and public health factors that influence disease transmission. In this manuscript, we denote the basic reproduction number as $R_c$ in the manuscript, whereas $R_0$ refers to values reported in the literature.

## References


[1] Chowell G, Diaz-Duenas P, Miller JC, Alcazar-Velazco A, Hyman JM, Fenimore PW, et al. (2007) Estimation of the reproduction number of dengue fever from spatial epidemic data. Mathematical Biosciences 208:571-89.

[2] Peña-García VH, Christofferson RC (2019) Correlation of the basic reproduction number (R 0) and eco-environmental variables in Colombian municipalities with chikungunya outbreaks during 2014-2016. PLoS Neglected Tropical Diseases 1:e0007878.

[3] Enduri MK, Jolad S (2017) Estimation of reproduction number and non stationary spectral analysis of dengue epidemic. Mathematical Biosciences 288:140-8.

[4] Sanusi W, Badwi N, Zaki A, Sidjara S, Sari N, Pratama MI, et al. (2021) Analysis and simulation of SIRS model for dengue fever transmission in South Sulawesi, Indonesia. Journal of Applied Mathematics 2021:2918080.

[5] Ong J, Soh S, Ho SH, Seah A, Dickens BS, Tan KW, et al. (2022) Fine-scale estimation of effective reproduction numbers for dengue surveillance. PLoS Computational Biology 18:e1009791.

[6] Rodrigues HS, Monteiro MT, Torres DF, Zinober A (2012) Dengue disease, basic reproduction number and control. International Journal of Computer Mathematics 89:334-46.
