# Supplementary material for: Modeling treatment and temperature effects on dengue transmission at the division level in Bangladesh
Source: PLoS One. 2026 May 15;21(5):e0348077. doi: 10.1371/journal.pone.0348077 (PMC13178928; doi:10.1371/journal.pone.0348077)
Supplement: S3 Table — (PDF) [file pone.0348077.s005.pdf]

Table S3. Sensitivity indices of R_c to the parameters for the model

Parameter	Parameter values	Sensitivity indices
β_h	0.0328	$+1$
μ_h	0.0143	-0.000162
γ_h	0.32883	-0.7824
τ_h	0.03940	-0.2157
β_v	0.0100	+1
μ_v	0.00320	-1
b	0.2000	+2

This table presents the sensitivity indices of the R_c with respect to various model parameters. Parameters β_h, β_v, and b have the highest positive influence on R_c, while μ_v and γ_h show pronounced negative effects. These findings highlight the key factors that most significantly impact disease transmission and control.
