# Supplementary material for: Modeling treatment and temperature effects on dengue transmission at the division level in Bangladesh
Source: PLoS One. 2026 May 15;21(5):e0348077. doi: 10.1371/journal.pone.0348077 (PMC13178928; doi:10.1371/journal.pone.0348077)
Supplement: S4 Table — (PDF) [file pone.0348077.s006.pdf]

Table S4: Estimated parameters, associated errors, R^2 and R_c.

Administrative Name	I_h^0	Estimated parameters						Error metrics			R^2	R_c
		τ_h	β_h	γ_h	β_v	b	ρ	MAE	RMSE	nRMSE		
Dhaka Metrop.	18	0.95	0.37	2.93	0.96	3.83	0.01	157.82	211.73	0.072	0.95	1.6937
Dhaka Division	24	0.94	0.97	2.90	0.74	3.87	0.0073	238.15	326.82	0.073	0.95	1.7210
Mymensingh Div.	5	0.90	0.79	2.14	0.23	3.61	0.001	13.120	19.73	0.073	0.94	1.7318
Chittagong Div.	29	0.99	0.52	2.99	0.99	3.98	0.0024	80.24	108.78	0.10	0.88	1.6774
Khulna Division	2	0.82	0.83	2.75	0.33	3.82	0.0028	41.33	62.60	0.07	0.94	1.7472
Rajshahi Division	1	0.96	0.99	0.0008	0.105	3.99	0.0002	24.74	51.81	0.11	0.81	2.2997
Rangpur Division	1	0.87	0.18	0.14	0.86	3.38	0.0001	7.24	13.76	0.075	0.913	2.7019
Barisal Division	6	0.84	0.69	2.79	0.39	2.76	0.0048	104.35	227.26	0.23	0.20	1.6867
Sylhet Division	1	0.19	0.61	2.99	0.82	2.15	0.0001	2.35	3.87	0.10	0.86	1.7910

For the computation of the basic reproduction number, the human population N_h for each division and Dhaka Metropolitan was taken from Table S1, while the parameters N_v, μ_h, and μ_v were obtained from Table 2 (second column). Table S4 shows that dengue transmission dynamics across Bangladesh highlights treatment (τ_h) as a pivotal factor in outbreak control. Regions with higher treatment levels, such as Dhaka Metropolitan ($\tau_h = 0.95$) and Chittagong ($\tau_h = 0.99$), exhibited strong model fits ($R^2 \approx 0.9 - 0.95$) and relatively low $R_c \approx 1.7$. Indicating effective epidemic suppression. In contrast, regions with weaker treatment contributions, including Rangpur and Barisal, showed larger R_c values and reduced predictive accuracy, underscoring that inadequate treatment hampers outbreak control.

An exception was observed in Rajshahi Division, where the treatment parameter was high ($\tau_h = 0.96$) but the recovery rate was nearly zero ($\gamma_h \approx 0.0008$). This combination led to a poor model fit ($R^2 = 0.81$) and elevated reproduction number ($R_c = 2.30$), demonstrating that treatment alone is not sufficient without adequate recovery dynamics. This highlights the importance of health system capacity and supportive care in conjunction with treatment interventions.

Overall, our findings confirm that enhancing supportive treatment significantly reduces infection peaks and delays epidemic spread, while regional variations reveal the need for locally tailored strategies. These results underscore the importance of integrating treatment-focused interventions with temperature-sensitive transmission models to guide proactive dengue control policies in Bangladesh.
