# Supplementary material for: Modeling treatment and temperature effects on dengue transmission at the division level in Bangladesh
Source: PLoS One. 2026 May 15;21(5):e0348077. doi: 10.1371/journal.pone.0348077 (PMC13178928; doi:10.1371/journal.pone.0348077)
Supplement: S5 Table — (PDF) [file pone.0348077.s007.pdf]

Table S5: Summary of literature on dengue in Bangladesh, highlighting treatment-related aspects, biting behaviour, temperature influences, and references. While prior studies emphasize the absence of specific antiviral therapies and the reliance on supportive care, none directly quantify the impact of treatment on dengue transmission dynamics. Similarly, although climatic factors such as temperature, rainfall, and humidity are frequently linked to mosquito abundance and transmission risk, these effects are rarely integrated with treatment in a unified modeling framework. Addressing this gap, our study develops a metapopulation model that explicitly incorporates both treatment and temperature to examine dengue transmission and control at the provincial level in Bangladesh.

Paper Title	Treatment-related aspects	Biting and Temperature-related aspects	Ref.
Understanding dengue outbreaks in Rajshahi district, Bangladesh: A comprehensive case study	As there is no specific antiviral treatment exists for dengue, patient care remains supportive. Identifying the specific dengue serotype is considered crucial for effective treatment and disease management. The study acknowledges the healthcare professionals at Rajshahi Medical College Hospital for their "exceptional care in treating patients."	Dengue is transmitted by daytime bites from infected female *Aedes aegypti* and *Aedes albopictus* mosquitoes. Favorable breeding conditions for these mosquitoes in Bangladesh exacerbate the threat. Regional variations in dengue serotypes might be influenced by local climatic conditions.	[1]
Circulating dengue virus serotypes, demographics, and epidemiology in the 2023 dengue outbreak in Chittagong, Bangladesh	The vast majority of patients (99.2%) did not receive any specific medication during recovery. The study mentions the potential for prophylactic interventions for dengue in Bangladesh.	The higher prevalence of dengue in males is suggested to be due to behavioural patterns, such as spending more time outdoors at dawn and in the evening, which increases exposure to mosquito vectors.	[2]
Dengue Virus Serotype 2 Cosmopolitan C Genotype Reemerges with a New Subclade in Southwest Region of Bangladesh	Dengue can manifest in a wide clinical spectrum, ranging from mild fever to severe and sometimes fatal disease. A secondary infection with a different serotype or genotype can increase disease severity due to antibody-dependent enhancement (ADE).	This paper focuses on genomic surveillance and does not provide specific information on biting habits or temperature.	[3]
Recent outbreak of dengue in Bangladesh: A threat to public health	There is no specific cure for dengue; treatment for severe cases may involve supportive care like blood transfusions and intravenous fluids. A safe and effective dengue vaccine would need to be tetravalent, offering protection against all four serotypes simultaneously.	The disease is spread by the bite of an infected *Aedes* mosquito. Bangladesh's year-round warm temperatures create an ideal environment for mosquito reproduction and virus replication. The spread of dengue is being accelerated due to global drivers such as rising temperatures. The	[4]

		severity of dengue may decrease during the winter season.	
A perspective on the worst ever dengue outbreak 2023 in Bangladesh: What makes this old enemy so deadly, and how can we combat it?	There is currently no specific cure or treatment for dengue; management is supportive and focuses on controlling symptoms like fever and fluid loss. Severe cases may require hospitalization for blood transfusions and intravenous fluids. The paper highlights a successful trial of the TV005 tetravalent dengue vaccine in Dhaka as a promising step.	The virus is transmitted by the *Aedes* mosquito. The country's tropical temperature and monsoon rains are conducive to mosquito breeding. Climate change has led to higher temperatures and increased rainfall, helping mosquito vectors thrive.	[5]
Epidemiology and evolution of dengue outbreaks in Bangladesh (2020-2023)	The evolution of the dengue virus highlights the need to develop strain-specific drugs and vaccines.	Dengue is transmitted to humans through the bites of infected mosquitoes, primarily *Aedes aegypti*. The rise in cases is linked to unusually high temperatures, high humidity, and sporadic rainfall, which increase mosquito populations. A positive correlation has been found between dengue infections and both temperature and rainfall.	[6]
Dengue epidemic in a non-endemic zone of Bangladesh: Clinical and laboratory profiles of patients	Patients in the study received standard care according to WHO guidelines. Prompt therapeutic management is crucial during an epidemic. Severe clinical bleeding may necessitate a blood transfusion.	The virus is transmitted by infected *Aedes aegypti* and occasionally *Aedes albopictus* mosquitoes. Factors like climate change, humid weather, and the rainy season contribute to the availability of mosquito breeding sources.	[7]
Evolving epidemiology, clinical features, and genotyping of dengue outbreaks in Bangladesh, 2000–2024: a systematic review	Dengue virus-specific antivirals are currently unavailable, and dengue vaccines are not approved for use in Bangladesh. A recent trial of the TV005 tetravalent vaccine is noted as a promising development.	The virus is transmitted by *Aedes spp.* mosquitoes. The optimal temperature for the vector's reproduction is between 23°C and 29°C. During peak dengue months in Bangladesh (August to November), the average temperature ranges from 26°C to 31°C, supporting the seasonal rise in cases. Climate change, rising temperatures, and prolonged monsoon seasons are fuelling larger outbreaks.	[8]

Knowledge, Attitude and Practices Towards Dengue Fever Among Slum Dwellers: A Case Study in Dhaka City, Bangladesh	The study notes there is no effective vaccination for dengue fever. When symptoms appear, a majority of respondents (57%) would go to a hospital for testing and treatment.	About 39% of slum dwellers surveyed knew that the *Aedes* mosquito typically bites in the early morning and late evening. The paper mentions that climate change could lead to a significant dengue outbreak in Dhaka.	[9]
Concurrent transmission of Zika virus during the 2023 dengue outbreak in Dhaka, Bangladesh	There are no specific vaccines or medications to prevent Zika virus (ZIKV), which complicates diagnosis and treatment when its symptoms overlap with dengue.	ZIKV was isolated from a pool of *Aedes* mosquitoes, and the urban transmission cycle involves *Ae. aegypti*.	[10]
Twenty-two years of dengue outbreaks in Bangladesh: epidemiology, clinical spectrum, serotypes, and future disease risk	No specific treatment is widely available, and a licensed vaccine is not widely accessible. Improved clinical management has helped lower the case fatality rate in recent years. Severe cases often need ICU support, which is mainly available in major cities	Public awareness of the biting behavior of vector mosquitoes remains limited, while the survival of *Aedes* mosquitoes is highly sensitive to temperature, with an optimum range between 23°C and 29°C. Climate change could potentially extend the dengue transmission season in Bangladesh to be all year round by the end of the century.	[11]

References

[1] Sumon MM, Jubair M, Tony SR, Islam MJ, Paul DK, Al Shaharia M, et al. (2024). Understanding dengue outbreaks in Rajshahi district, Bangladesh: a comprehensive case study. IDCases 37:e02032.

[2] Rob MA, Hossain M, Sattar MA, Ahmed IU, Chowdhury AF, Mehedi HH, et al. (2024) Circulating dengue virus serotypes, demographics, and epidemiology in the 2023 dengue outbreak in Chittagong, Bangladesh. European Journal of Microbiology and Immunology 14:272-9.

[3] Setu MA, Das PK, Ahammed T, Saha S, Hasan A, Kumar PS, et al. (2023) Dengue virus serotype 2 cosmopolitan C genotype reemerges with a new subclade in southwest region of Bangladesh. *medRxiv* 28:2023-12.

[4] Bhowmik KK, Ferdous J, Baral PK, Islam MS (2023) Recent outbreak of dengue in Bangladesh: a threat to public health. Health Science Reports 6:e1210.

[5] Sarker R, Roknuzzaman AS, Emon FA, Dewan SM, Hossain MJ, Islam MR (2024) A perspective on the worst ever dengue outbreak 2023 in Bangladesh: What makes this old enemy so deadly, and how can we combat it?. Health Science Reports 7:e2077.

[6] Hasan M, Islam S (2024) Epidemiology and evolution of dengue outbreaks in Bangladesh (2020–2023). One Health Bulletin 4:60-8.

[7] Rafi A, Mousumi AN, Ahmed R, Chowdhury RH, Wadood A, Hossain G (2020) Dengue epidemic in a non-endemic zone of Bangladesh: clinical and laboratory profiles of patients. PLoS Neglected Tropical Diseases 14:e0008567.

[8] Sharif N, Opu RR, Saha T, Masud AI, Naim J, Alsharif KF, et al. (2024) Evolving epidemiology, clinical features, and genotyping of dengue outbreaks in Bangladesh, 2000–2024: a systematic review. Frontiers in Microbiology 15:1481418.

[9] Rahman MM, Tanni KN, Roy T, Islam MR, Rumi MA, Sakib MS, et al. (2023) Knowledge, attitude and practices towards dengue fever among slum dwellers: a case study in Dhaka City, Bangladesh. International Journal of Public Health 68:1605364.

[10] Hasan A, Hossain MM, Zamil MF, Trina AT, Hossain MS, Kumkum A, et al. (2025) Concurrent transmission of Zika virus during the 2023 dengue outbreak in Dhaka, Bangladesh. PLoS Neglected Tropical Diseases 19:e0012866.

[11] Hossain MS, Noman AA, Mamun SM, Mosabbir AA (2023) Twenty-two years of dengue outbreaks in Bangladesh: epidemiology, clinical spectrum, serotypes, and future disease risks. Tropical Medicine and Health 51:1-4.
