# Supplementary material for: Modeling treatment and temperature effects on dengue transmission at the division level in Bangladesh
Source: PLoS One. 2026 May 15;21(5):e0348077. doi: 10.1371/journal.pone.0348077 (PMC13178928; doi:10.1371/journal.pone.0348077)
Supplement: S6 Table — (PDF) [file pone.0348077.s008.pdf]

Table S6. Estimated reproduction numbers $\boldsymbol{R_c}$ for different divisions of Bangladesh.

**Name of Division**	$\boldsymbol{R_c}$**(using estimated parameters in Table S4)**	$\boldsymbol{R_c}$**(For column 2 of Table 2)**	$\boldsymbol{R_c}$**(For column 6 of Table 2)**
Dhaka Metrop.	1.6937	2.4448	251.6561
Dhaka Division	1.7210	1.1917	305.1603
Mymensing Division	1.7318	4.3098	157.6854
Chittanganj Division	1.6774	1.5869	136.4405
Khulna Division	1.7472	3.0252	159.4446
Rajshahi Division	2.2997	2.5888	83.6384
Rangpur Division	2.7019	2.9919	227.1474
Barishal Division	1.6867	5.7900	62.8064
Sylhet Division	1.7910	4.7748	128.8553

The reproduction numbers reported in Table S6 were computed using the division-specific human population sizes N_h obtained from Table S1. Specifically, the first column in **Table S6** of estimates is based on the fitted parameter values presented in Table S4. The third and fourth columns $\boldsymbol{R_c}$ **in Table S6** were obtained using the parameter sets reported in Columns 2 and 6 of Table 2, respectively, combined with the corresponding division-wise population sizes.

We observe that the values of $\boldsymbol{R_c}$ obtained using literature-based parameter sets (fourth columns $\boldsymbol{R_c}$ **in Table S6**) are substantially larger than those obtained from the fitted parameters. This discrepancy likely reflects differences in parameter assumptions and underscores the importance of data-driven estimation to obtain realistic, region-specific estimates of transmission potential.
