# Supplementary material for: Modeling treatment and temperature effects on dengue transmission at the division level in Bangladesh
Source: PLoS One. 2026 May 15;21(5):e0348077. doi: 10.1371/journal.pone.0348077 (PMC13178928; doi:10.1371/journal.pone.0348077)
Supplement: Attachment — Submitted filename: Recommendation.pdf [file pone.0348077.s009.pdf]

Manuscript Evaluation Report

Manuscript Title

Modeling Treatment and Temperature Effects on Dengue Transmission at the Division Level in Bangladesh.

Summary of the Manuscript

This study provides a valuable contribution by integrating treatment access and efficacy with temperature-dependent mosquito biting dynamics in a dengue transmission model for Bangladesh. The coupling of a human SITRS framework with a mosquito SI model and the distinction between natural recovery and supportive care offer a realistic representation of healthcare disparities. The analytical treatment and calibration with surveillance and temperature data strengthen the study, while the results highlight the important role of timely treatment in reducing epidemic intensity. Overall, the framework is well motivated and has strong potential for application in climate-sensitive epidemic preparedness.

While the study makes a valuable contribution, several aspects of the presentation and analysis require careful revision to improve clarity and consistency. Addressing these issues would further strengthen the rigor and impact of the work.

Minor Comments

1. The manuscript contains several spelling inconsistencies (e.g. suppohomorting) that should be carefully reviewed and corrected throughout.
2. The reference numbering does not appear to follow a consistent order throughout the manuscript. Please review and revise the placement of references to ensure proper sequencing.
3. A careful check of the reference list is recommended, as certain references seem to appear repeatedly.
4. The parameter τ_h is inconsistently described as a specified time interval, a disease progression rate, and a treatment rate in different sections. Clarification and revision are requested to ensure consistency.

5. Please check the formatting of columns 6 and 7 of table 2, as they appear to be merged.
6. Please carefully check the x-tick labels in Fig. 2, Panel B, as they may require correction.
7. Please carefully revise the theorem (Theorem 3) statement to ensure consistency between the stability condition and the biological interpretation.

Major Comments

1. Given that Fig. 8 depicts scenarios for human infections (I_h). Clarification is requested on how conclusions regarding infection dynamics in both human and mosquito populations were drawn, and what analyses were conducted to assess the role of mosquito control under epidemic conditions.
2. Could you please clarify how the analysis presented in Fig. 9 supports conclusions regarding infection dynamics in the mosquito population? While treatment may indirectly reduce mosquito infections by lowering human infectiousness, it would be helpful to explain how such conclusions were drawn in the absence of explicit vector control interventions or mosquito-targeted measures in the model.
3. The temperature-dependent biting rate function b(T) is directly adopted from reference [43]. Could you please clarify the rationale for applying this formulation in the Bangladesh context? In particular, clarification is requested regarding the suitability of the adopted formulation for Bangladesh-specific conditions and whether any validation or sensitivity analyses were performed.
4. Figure 5, Panel A is described as using the baseline parameter values listed in Table 2; however, Table 2 reports a basic reproduction number $R_c = 3.0456$ for these parameter values, whereas Figure 5 indicates $R_c < 1$. Could you please clarify this apparent discrepancy and explain how the condition $R_c < 1$ was obtained in Figure 5?
5. The figure description (Fig. 6) appears to be inconsistent with the content shown in the figure. Clarification or revision would be appreciated to ensure consistency between the figure and its description.
6. While sensitivity analyses of epidemiological parameters in human and vector classes are presented, the inclusion of an LHS–PRCC approach could further account for parameter uncertainty.
7. Please consider placing figures within their corresponding sections in the main manuscript and integrating the correlation figures from the supporting file where appropriate.
8. The manuscript would benefit from engagement with additional literature on dengue transmission, treatment-based intervention modeling, and uncertainty quantification, as well as broader infectious disease modelling studies on host–vector dynamics, threshold analysis, and control strategies.

a) Cost-effectiveness of dengue control strategies in Bangladesh: An optimal control and ACER-ICER analysis. https://doi.org/10.1016/j.actatropica.2025.107587

b) Optimal control analysis of COVID-19 transmission model with physical distance and treatment. http://dx.doi.org/10.26855/abr.2022.12.001.

c) Analysis of a Data-Driven Vector-Borne Dengue Transmission Model for a Tropical Environment in Bangladesh. https://doi.org/10.1155/2024/2959770

d) Stochastic analysis of Mpox epidemiology with vaccination strategies and environmental persistence. https://doi.org/10.1038/s41598-025-28135-x

Recommendation

I recommend acceptance of the manuscript after the authors have adequately addressed the major and minor issues outlined above. With these revisions, the manuscript will be substantially strengthened and better positioned to make a meaningful contribution to the field.
