# Supplementary material for: Modeling treatment and temperature effects on dengue transmission at the division level in Bangladesh
Source: PLoS One. 2026 May 15;21(5):e0348077. doi: 10.1371/journal.pone.0348077 (PMC13178928; doi:10.1371/journal.pone.0348077)
Supplement: Attachment — Submitted filename: Recommendations.pdf [file pone.0348077.s011.pdf]

Manuscript Evaluation Report

Manuscript Title

Modeling Treatment and Temperature Effects on Dengue Transmission at the Division Level in Bangladesh.

General Evaluation of the Manuscript

The study contributes useful insights; however, multiple aspects of the presentation and analysis need careful revision to improve clarity and maintain consistency throughout. Moreover, it is not good practice to claim that an issue has been revised or corrected when the revised manuscript does not clearly reflect the stated changes (e.g., in theorem statements, figure captions, or axis labels). All reported corrections should be explicitly implemented and presented consistently across the text and figures to avoid ambiguity and reader confusion.

Comments

Comment 1. **Theorem 3** remains inconsistent: it states global stability for $R_c^{NG} \leq 1$ but interprets the equilibrium as "positively endemic." That conclusion should not follow from $R_c^{NG} \leq 1$. Also, the authors claim this has been corrected, but the correction is not reflected in the current text—please revise the theorem statement and its interpretation accordingly.

Comment 2. The **Figure 2** still contains an incorrect year label (e.g., "01-01-2024" appears between 2021 and 2022 in the zoomed panel), indicating the axis labeling issue remains unresolved. Despite the authors' claim of revision, the error remains visible in the current figure—please fix the date labels and ensure chronological consistency across panels.

Comment 3. The clarification remains inconsistent in **Figure 5**: the response states Panel A uses modified parameters to achieve $R_c^{NG} < 1$ and Panel B uses the baseline Table 2 values ($R_c^{NG} = 3.0456$), but the updated Figure 5 caption reverses this (Panel A baseline; Panel B modified). Please ensure the caption and response are consistent, and confirm which panel corresponds to the baseline case ($R_c^{NG} = 3.0456$) and which uses modified parameters to obtain $R_c^{NG} < 1$.

Comment 4. The caption is inconsistent in **Figure 8**: it claims high sensitivity to τ_h but also states the outcome has minimal response to τ_h (likely meant δ_h). In addition, the severity-level magnitude comparison is unclear due to seemingly inconsistent colorbar scaling—please standardize the scales and revise the caption.

Recommendation

I can recommend acceptance only if the authors satisfactorily address the issues noted above and ensure that all claimed corrections are clearly implemented in the revised manuscript. It is not good practice to state that problems have been corrected when the revision does not reflect those changes. If these concerns remain unresolved, I cannot support acceptance.
