# Supplementary material for: Modeling treatment and temperature effects on dengue transmission at the division level in Bangladesh
Source: PLoS One. 2026 May 15;21(5):e0348077. doi: 10.1371/journal.pone.0348077 (PMC13178928; doi:10.1371/journal.pone.0348077)
Supplement: Attachment — Submitted filename: Response to Editor and Reviewers comments.pdf [file pone.0348077.s012.pdf]

PONE-D-25-62939

Title: *Modeling Treatment and Temperature Effects on Dengue Transmission at the Division Level in Bangladesh*

Journal: PLOS ONE

Dear Editor,

We sincerely thank you and the reviewers for providing us with the opportunity to revise our manuscript again. We greatly appreciate the thoughtful and constructive feedback, which have helped us strengthen the clarity, rigor, and scientific quality of our work.

In response to the comments, we have thoroughly revised the manuscript. All substantive changes have been clearly marked in **red text** in the revised document to facilitate review. Major improvements include:

- Clarifying Theorem 3.
- Revising **Figure 2,** Figure **5 and Figure 8.**
- Correcting all typographical, formatting, and referencing issues throughout the manuscript.

We have provided a **detailed point-by-point response** to each comment, demonstrating how each issue has been addressed in the revised manuscript.

We believe that these revisions have significantly improved our manuscript and that it now clearly communicates the scientific contributions and implications of our study. We respectfully submit the revised manuscript for your consideration and hope that it will be deemed suitable for publication in *PLOS ONE*.

Thank you for your consideration.

Sincerely,
Mr. Haridas Kumar Das and Professor Dr. Md Abdul Kuddus

Response to Editorial Requirements – PONE-D-25-62939

Thank you for your guidance regarding the submission requirements. We have addressed all points as follows:

Comment 1: Theorem 3 remains inconsistent: it states global stability form but interprets the equilibrium as "positively endemic." That conclusion should not follow from $R_c^{NG} \leq 1$. Also, the authors claim this has been corrected, but the correction is not reflected in the current text—please revise the theorem statement and its interpretation accordingly.

Response 1: Thank you so much for bringing that to our attention. The following is the updated statement and its proof.

Theorem 3. If $R_c > 1$, then the equilibrium status of dengue fever diseases is positively endemic, and equation system (1) exists and is in the global stage asymptotically stable in $D(Eq\ 3)$ by assuming that,

$$S_h^{**} = N_h,\ S_v^{**} = \frac{A}{\mu_V},\ \ \mu_h = \frac{\beta_v b}{N_h}\frac{(\mu_h+\gamma_h+\theta_h+\tau_h+\delta_h)}{r},\ \ \mu_v = \frac{r\beta_h b}{(\mu_h+\gamma_h+\theta_h+\tau_h+\delta_h)}S_v^{**}, \tag{10}$$

where $r = \frac{\beta_h b}{N_h}$, The mosquito population mortality rate is represented by μ_v, the number b indicates the rate of possibly infectious mosquito bites, $\beta_h b$ expresses the interaction capability between humans and mosquitoes as the vector, and the number of people in the population is likely equivalent to the number of possible cases of dengue disease.

Proof. We considered the Lyapunov function [48] *of the form in*

$$V(t) = (S_h - S_h^{**} lnS_h) + I_h + T_h + R_h + \frac{(\mu_h+\gamma_h+\theta_h+\tau_h+\delta_h)}{rS_v^{**}}(S_v - S_v^{**} lnS_v) + \frac{\mu_h+\gamma_h+\theta_h+\tau_h+\delta_h}{rS_v^{**}} I_v. \tag{11}$$

In order to satisfy equation (11), the derivative of $V(t)$ with respect to time t,

$$\dot{V}(t) = \dot{S_h}\left(1 - \frac{S_h^{**}}{S_h}\right) + \dot{I_h} + \dot{T_h} + \dot{R_h} + \dot{S_v}\left(1 - \frac{S_v^{**}}{S_v}\right)\left(\frac{\mu_h+\gamma_h+\theta_h+\tau_h+\delta_h}{rS_v^{**}}\right) + \dot{I_v}\ \frac{\mu_h+\gamma_h+\theta_h+\tau_h+\delta_h}{rS_v^{**}}$$
$$= \mu_h N_h\left(1 - \frac{S_h^{**}}{S_h}\right) - \frac{\beta_h b}{N_h} I_v S_h\left(1 - \frac{S_h^{**}}{S_h}\right) - \mu_h S_h\left(1 - \frac{S_h^{**}}{S_h}\right) + \theta_h R_h\left(1 - \frac{S_h^{**}}{S_h}\right) + \frac{\beta_h b}{N_h} I_v S_h - (\mu_h + \gamma_h + \tau_h) I_h$$
$$+ \tau_h I_h - \delta_h T_h - \mu_h T_h + \gamma_h I_h - (\mu_h + \theta_h) R_h + \delta_h T_h + \left(\frac{\mu_h+\gamma_h+\theta_h+\tau_h+\delta_h}{rS_v^{**}}\right) A\left(1 - \frac{S_v^{**}}{S_v}\right)$$
$$- \left(\frac{\mu_h+\gamma_h+\theta_h+\tau_h+\delta_h}{rS_v^{**}}\right)\frac{\beta_v b}{N_h} I_h S_v\left(1 - \frac{S_v^{**}}{S_v}\right) - \left(\frac{\mu_h+\gamma_h+\theta_h+\tau_h+\delta_h}{rS_v^{**}}\right)\mu_v S_v\left(1 - \frac{S_v^{**}}{S_v}\right) + \left(\frac{\mu_h+\gamma_h+\theta_h+\tau_h+\delta_h}{rS_v^{**}}\right)\frac{\beta_v b}{N_h} I_h S_v -$$
$$\left(\frac{\mu_h+\gamma_h+\theta_h+\tau_h+\delta_h}{rS_v^{**}}\right)\mu_v I_v$$
$$= \mu_h N_h\left(1 - \frac{S_h^{**}}{S_h}\right) + \theta_h R_h - \theta_h R_h\left(\frac{S_h^{**}}{S_h}\right) - \mu_h S_h\left(1 - \frac{S_h^{**}}{S_h}\right) - \frac{\beta_h b}{N_h} I_v S_h - \frac{\beta_h b}{N_h} I_v S_h^{**} + \frac{\beta_h b}{N_h} I_v S_h - \mu_h I_h -$$
$$\mu_h T_h - \mu_h R_h - \theta_h R_h + A\left(\frac{\mu_h+\gamma_h+\theta_h+\tau_h+\delta_h}{rS_v^{**}}\right) - A\left(\frac{\mu_h+\gamma_h+\theta_h+\tau_h+\delta_h}{rS_v}\right) - \frac{\beta_v b}{N_h} I_h S_v\left(\frac{\mu_h+\gamma_h+\theta_h+\tau_h+\delta_h}{rS_v^{**}}\right) +$$
$$\frac{\beta_v b}{N_h} I_h\left(\frac{\mu_h+\gamma_h+\theta_h+\tau_h+\delta_h}{r}\right) - \left(\frac{\mu_h+\gamma_h+\theta_h+\tau_h+\delta_h}{r}\right)\frac{\beta_h b}{N_h} I_h - \left(\frac{\mu_h+\gamma_h+\theta_h+\tau_h+\delta_h}{r}\right)\frac{\beta_h b}{N_h} I_v\left(\frac{S_v}{S_v^{**}}\right) -$$
$$\left(\frac{\mu_h+\gamma_h+\theta_h+\tau_h+\delta_h}{rS_v^{**}}\right)\mu_v I_v$$
$$= \mu_h N_h\left(1 - \frac{S_h^{**}}{S_h}\right) + \mu_h S_h^{**}\left(1 - \frac{S_h^{**}}{S_h}\right) - \theta_h R_h\left(\frac{S_h^{**}}{S_h}\right) - \mu_h R_h - \frac{\beta_h b}{N_h} I_v S_h^{**} - \mu_h I_h - \mu_h T_h +$$
$$A\left(\frac{\mu_h+\gamma_h+\theta_h+\tau_h+\delta_h}{rS_v^{**}}\right) - A\left(\frac{\mu_h+\gamma_h+\theta_h+\tau_h+\delta_h}{rS_v}\right) + \frac{\beta_v b}{N_h} I_h\left(\frac{\mu_h+\gamma_h+\theta_h+\tau_h+\delta_h}{r}\right) - \left(\frac{\mu_h+\gamma_h+\theta_h+\tau_h+\delta_h}{r}\right)\left(\frac{S_v}{S_v^{**}}\right)\mu_v +$$
$$\mu_v\left(\frac{\mu_h+\gamma_h+\theta_h+\tau_h+\delta_h}{r}\right) - \left(\frac{\mu_h+\gamma_h+\theta_h+\tau_h+\delta_h}{rS_v^{**}}\right)\mu_v I_v.$$

(12)

Substituting equation (10) into equation (12), we can find

$$= \mu_h N_h \left(2 - \frac{S_h^{**}}{S_h} - \frac{S_h}{S_h^{**}}\right) - \theta_h R_h \left(\frac{S_h^{**}}{S_h}\right) - \mu_h R_h + \left(\frac{\beta_h b}{N_h} S_h^{**} - \left(\frac{\mu_h+\gamma_h+\theta_h+\tau_h+\delta_h}{r S_v^{**}}\right)\mu_v\right) I_v - \mu_h T_h + \left(\frac{\beta_h b}{N_h}\left(\frac{\mu_h+\gamma_h+\theta_h+\tau_h+\delta_h}{r}\right) - \mu_h\right) I_h + \left(\frac{\mu_h+\gamma_h+\theta_h+\tau_h+\delta_h}{r S_v^{**}}\right)\mu_v S_v^{**} - \left(\frac{\mu_h+\gamma_h+\theta_h+\tau_h+\delta_h}{r S_v}\right)\mu_v S_v^{**} + \mu_v\left(\frac{\mu_h+\gamma_h+\theta_h+\tau_h+\delta_h}{r}\right) - \left(\frac{\mu_h+\gamma_h+\theta_h+\tau_h+\delta_h}{r}\right)\left(\frac{S_v}{S_v^{**}}\right)\mu_v$$

$$= \mu_h N_h \left(2 - \frac{S_h^{**}}{S_h} - \frac{S_h}{S_h^{**}}\right) - \theta_h R_h \left(\frac{S_h^{**}}{S_h}\right) - \mu_h R_h - \mu_h T_h + \frac{\mu_v}{r} 2(\mu_h + \gamma_h + \theta_h + \tau_h + \delta_h) - \mu_v\left(\frac{\mu_h+\gamma_h+\theta_h+\tau_h+\delta_h}{r}\right)\left(\frac{S_v}{S_v^{**}}\right) - -\mu_v\left(\frac{\mu_h+\gamma_h+\theta_h+\tau_h+\delta_h}{r}\right)\left(\frac{S_v^{**}}{S_v}\right)\mu_h N_h \left(2 - \frac{S_h^{**}}{S_h} - \frac{S_h}{S_h^{**}}\right) - \mu_v\left(\frac{\mu_h+\gamma_h+\theta_h+\tau_h+\delta_h}{r}\right)\left(2 - \frac{S_h^{**}}{S_h} - \frac{S_h}{S_h^{**}}\right) - \theta_h R_h$$

$$= -\mu_h N_h \left(\frac{\left(S_h - S_h^{**}\right)^2}{S_h S_h^{**}}\right) - \mu_v\left(\frac{\mu_h+\gamma_h+\theta_h+\tau_h+\delta_h}{r}\right)\left(\frac{\left(S_h - S_h^{**}\right)^2}{S_h S_h^{**}}\right) - \theta_h R_h \left(\frac{S_h^{**}}{S_h}\right) - \mu_h R_h - \mu_h T_h.$$

(13)

Equation (13) shows that $\dot{V}(t) \leq 0$ for all $S_h^{**}, I_h^{**}, T_h^{**}, R_h^{**}, S_v^{**}, I_v^{**} \epsilon D, S_h^{**}, I_h^{**}, T_h^{**}, R_h^{**}, S_v^{**}, I_v^{**}$ and $\dot{V}(t) = 0$ for $S_h = S_h^{**},\ I_h = I,\ T_h = T_h^{**},\ R_h = R_h^{**},\ S_v = S_v^{**},\ and\ I_v = I_v^{**}$. Then the endemic equilibrium point P^{**} is a set of positive invariant of system (1) that is contained in

$$L = \{(S_h(t), I_h(t), T_h(t), R_h(t), S_v(t), I_v(t)), S_h = S_h^{**}, I_h = I, T_h = T_h^{**}, R_h = R_h^{**}, S_v = S_v^{**}, I_v = I_v^{**}\}.$$

Using the asymptotical stability theorem, positive endemic equilibrium P^{**} is globally asymptotically stable in $D(Eq\ 3)$. This proves Theorem 3.

The global stability theorem for the model SITRS in this stage tells if an individual is infected with dengue fever with $R_c > 1$; then, the individual will likely infect at least another individual. Thus, the dengue fever in this situation has been endemic, uncontrolled, and threatening for the human population within the region.

Comment 2: The Figure 2 still contains an incorrect year label (e.g., "01-01-2024" appears between 2021 and 2022 in the zoomed panel), indicating the axis labeling issue remains unresolved. Despite the authors' claim of revision, the error remains visible in the current figure—please fix the date labels and ensure chronological consistency across panels.

Response 2: Thank you so much for bringing that typo to our attention. We sincerely appreciate your careful review of Figure 2. The issue with the date labeling in Figure 2 has been fully corrected. The incorrect year label ("01-01-2024") has been replaced with the correct date, "01-01-2021," from the zoomed panel. Additionally, the x-axis labels have been revised to ensure chronological consistency across all panels. The updated Figure 2 is shown below:

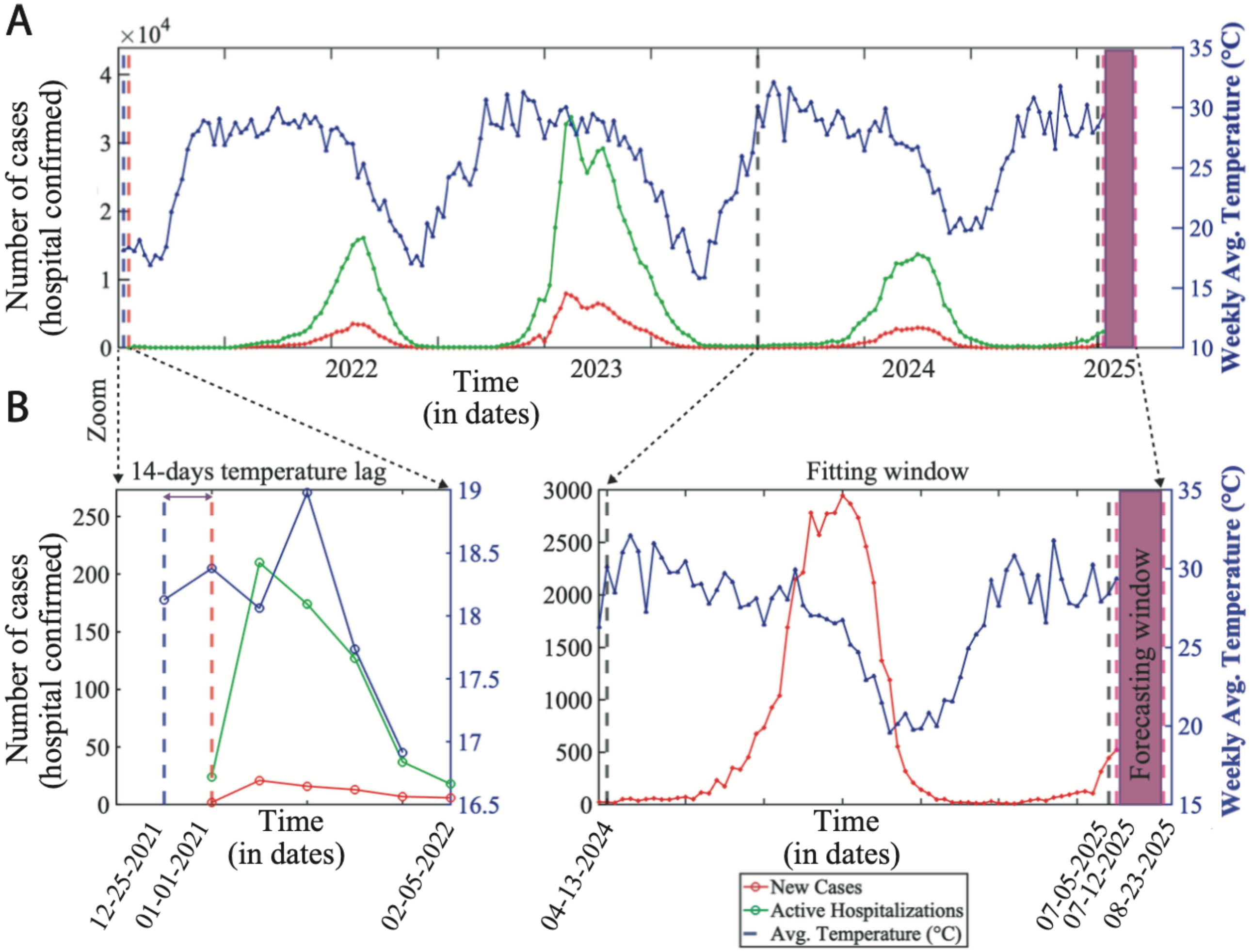

Figure 2: The Dhaka metropolitan area's weekly dengue incidences (hospital confirmed) and average temperature from 2021 to 2025.

In Figure 2, Panel A displays the time series of dengue cases that were reported in the Dhaka Metropolitan Area from January 1, 2022, to August 23, 2025. The lower-left part of Panel B displays a zoomed-in image of the model fit to reported dengue cases. The mosquito bite rate function $b(T)$ is defined using the average temperature($°C$) over the preceding 14 days. The right part of panel B provides a further zoomed view of the dataset divided into two phases: the fitting phase from April 13, 2024, to July 05, 2025 (EW 15, 2024 to EWS 27, 2025), and the forecasting phase from July 12, 2025, to August 23, 2025 (EW 28, 2025 to EW 34, 2025).

Comment 3: The clarification remains inconsistent in Figure 5: the response states Panel A uses modified parameters to achieve $R_c^{NG} < 1$ and Panel B uses the baseline Table 2 values $R_c^{NG} = 3.0456$, but the updated Figure 5 caption reverses this (Panel A baseline; Panel B modified). Please ensure the caption and response are consistent, and confirm which panel corresponds to the baseline case ($R_c^{NG} = 3.0456$) and which uses modified parameters to obtain $R_c^{NG} < 1$.

Response 3: Thank you for pointing out. I understand the difference pointed out between the caption and the answer provided in the figure for Figure 5. The problem was extensively investigated and addressed. The revised text now makes the following points clear below:

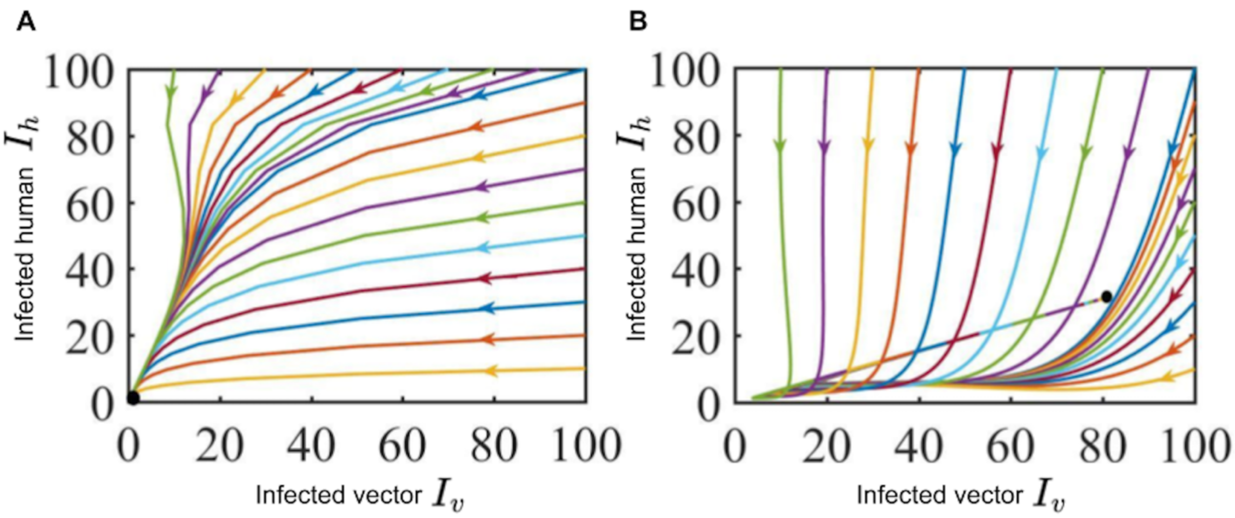

Figure 5. Impact of disease dynamics for two different parameter systems for 10 years. Panel A, using the baseline parameter values listed in Table 2, except that the human population is taken as ($N_h = 62700000$), the basic reproduction number is ($\boldsymbol{R_c = 0.8401 < 1}$). This indicates that the disease-free equilibrium is locally asymptotically stable and the infection eventually dies out over time. **Panel B,** for the modified parameter set ($\mu_h = 0.01, \gamma_h = 0.05, \tau_h = 0.07, \delta_h = 0.05, \mu_v = 0.02, \theta_h = 0.01, N_h = 15295319, N_v = 20080000, b = 0.07, \beta_h = 0.755, \beta_v = 0.855$), the reproduction number increases to ($\boldsymbol{R_c = 1.277 > 1}$). This implies that the disease-free equilibrium becomes unstable and the system converges to an endemic equilibrium where the infection persists in the population.

Comment 4: The caption is inconsistent in Figure 8: it claims high sensitivity to τ_h but also states the outcome has minimal response to τ_h (likely meant δ_h). In addition, the severity-level magnitude comparison is unclear due to seemingly inconsistent colorbar scaling—please standardize the scales and revise the caption.

Response 4: We acknowledge that the previous caption for Figure 8 was unclear, and we have made the necessary updates. The caption has been revised throughout and now accurately states "δ_h" instead of "high sensitivity to τ_h." Additionally, we have adjusted the colorbar scale to ensure it is consistent across all severity levels. To clarify, the updated caption distinguishes between responsiveness and parameter sensitivity.

Below are the revised details for Figure 8 and its caption:

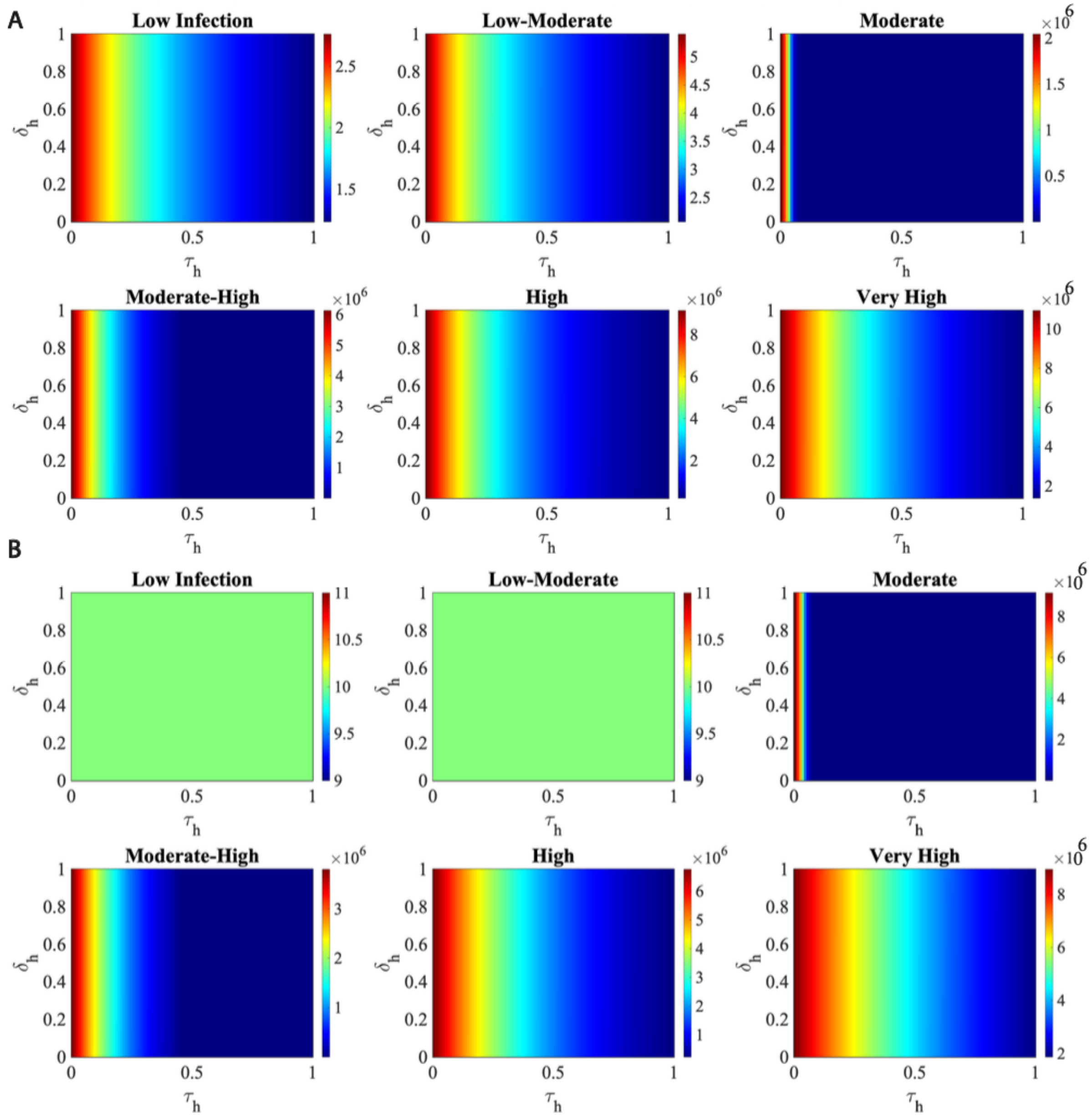

Figure 8. Heatmaps of peak simulated infection levels in humans and vectors for the six infection risk

scenarios with differing treatment and recovery rates.

Figure 8 shows a multidimensional sensitivity plot of the parameters τ_h and δ_h across the six levels of infection severity. In Panel A, there is a strong tilt to the parameter τ_h, where the changes to the parameter δ_h don't really do much to the outcome. As the parameter τ_h approaches zero, the peak outcome goes into the red, and then quickly drops back to the baseline as τ_h increases. The level of infection severity is what really makes the outcome spike, going from 2.5 to over $10 * 10^6$ from the level labeled "Low" to the level labeled "Very High." Panel B is a bit different for the vectors, where in the early stages of the infection, represented by the levels labeled "Low" and "Low-Moderate," the parameters don't seem to make much difference, where the heatmap is flat, but after the level labeled "Moderate," the sensitivity to the parameter τ_h is back, represented by the vertical line pattern, similar to Panel A in Figure 8. This could indicate a threshold effect, where the parameter τ_h has an effect only after a certain level of severity is reached. In both panels, the colors don't really change much in the vertical direction, indicating that the parameter τ_h is the driving force, where the parameter δ_h has little to no effect in the parameter space.

Extra Response: We have added a new table (Table S5) in the Appendix of the revised manuscript to provide division-wise estimates of the reproduction number ($\boldsymbol{R_c}$).

Table S5. Estimated reproduction numbers $\boldsymbol{R_c}$ for different divisions of Bangladesh.

**Name of Division**	**R_c(using estimated parameters in Table S4)**	**R_c(For column 2 of Table 2)**	**R_c(For column 6 of Table 2)**
Dhaka Metrop.	1.6937	2.4448	251.6561
Dhaka Division	1.7210	1.1917	305.1603
Mymensing Division	1.7318	4.3098	157.6854
Chittanganj Division	1.6774	1.5869	136.4405
Khulna Division	1.7472	3.0252	159.4446
Rajshahi Division	2.2997	2.5888	83.6384
Rangpur Division	2.7019	2.9919	227.1474
Barishal Division	1.6867	5.7900	62.8064
Sylhet Division	1.7910	4.7748	128.8553

The reproduction numbers reported in Table S5 were computed using the division-specific human population sizes N_h obtained from Table S1. Specifically, the first column in **Table S5** of estimates is based on the fitted parameter values presented in Table S4. The third and fourth columns $\boldsymbol{R_c}$ **in Table S5** were obtained using the parameter sets reported in Columns 2 and 6 of Table 2, respectively, combined with the corresponding division-wise population sizes.

We observe that the values of $\boldsymbol{R_c}$ obtained using literature-based parameter sets (fourth columns $\boldsymbol{R_c}$ **in Table S5**) are substantially larger than those obtained from the fitted parameters. This discrepancy likely reflects differences in parameter assumptions and underscores the importance of data-driven estimation to obtain realistic, region-specific estimates of transmission potential.
