# Supplementary material for: Modeling treatment and temperature effects on dengue transmission at the division level in Bangladesh
Source: PLoS One. 2026 May 15;21(5):e0348077. doi: 10.1371/journal.pone.0348077 (PMC13178928; doi:10.1371/journal.pone.0348077)
Supplement: Attachment — Submitted filename: Response to editor and reviewer comments.pdf [file pone.0348077.s010.pdf]

PONE-D-25-62939

Title: *Modeling Treatment and Temperature Effects on Dengue Transmission at the Division Level in Bangladesh*

Journal: PLOS ONE

Dear Editor,

We sincerely thank you and the reviewers for providing us with the opportunity to revise our manuscript. We greatly appreciate the thoughtful and constructive feedback provided by Reviewer #1 and Reviewer #2, which have helped us strengthen the clarity, rigor, and scientific quality of our work.

In response to the reviewers' comments, we have thoroughly revised the manuscript. All substantive changes have been clearly marked in **red text** in the revised document to facilitate review. Major improvements include:

- Clarifying definitions and correcting inconsistencies (e.g., parameter definitions, stability conditions, section nomenclature).
- Providing rigorous explanations for methods such as the sensitivity index and PRCC analysis, including biological interpretation.
- Clarifying initial conditions, scenario design, and temperature-dependent biting rate rationale in the Bangladesh context.
- Revising figures, tables, and their captions for accuracy and clarity, and repositioning figures appropriately within the corresponding sections.
- Expanding the literature review to include recent studies relevant to dengue transmission, intervention modeling, uncertainty quantification, and host–vector dynamics.
- Including additional analyses on partial rank correlation coefficient (PRCC) analysis to better capture parameter uncertainty.
- Correcting all typographical, formatting, and referencing issues throughout the manuscript.

We have provided a **detailed point-by-point response** to each reviewer comment, demonstrating how each issue has been addressed in the revised manuscript.

We believe that these revisions have significantly improved our manuscript and that it now clearly communicates the scientific contributions and implications of our study. We respectfully submit the revised manuscript for your consideration and hope that it will be deemed suitable for publication in *PLOS ONE*.

Thank you for your consideration.

Sincerely,
Mr. Haridas Kumar Das and Professor Dr. Md Abdul Kuddus

Response to Editorial Requirements – PONE-D-25-62939

Thank you for your guidance regarding the submission requirements. I have addressed all points as follows:

1. **Manuscript formatting and style:** The revised manuscript has been updated to comply with PLOS ONE's style requirements, including file naming conventions. We will be happy to work closely with the production team to ensure that any additional formatting needs are addressed.

2. **Code sharing:** All author-generated code underlying the findings **will be made publicly available** in accordance with PLOS ONE's guidelines to ensure reproducibility and facilitate reuse.

3. **Data repository:** All links to the dataset have been included in the main text and the Data Availability Statement.

4. **Figure references:** Figure 9 is now explicitly referred to in the text to ensure proper linking during production.

5. **Figure 1 copyright:** Thank you for your suggestion. Figure 1 has been updated and is shown below for your reference.

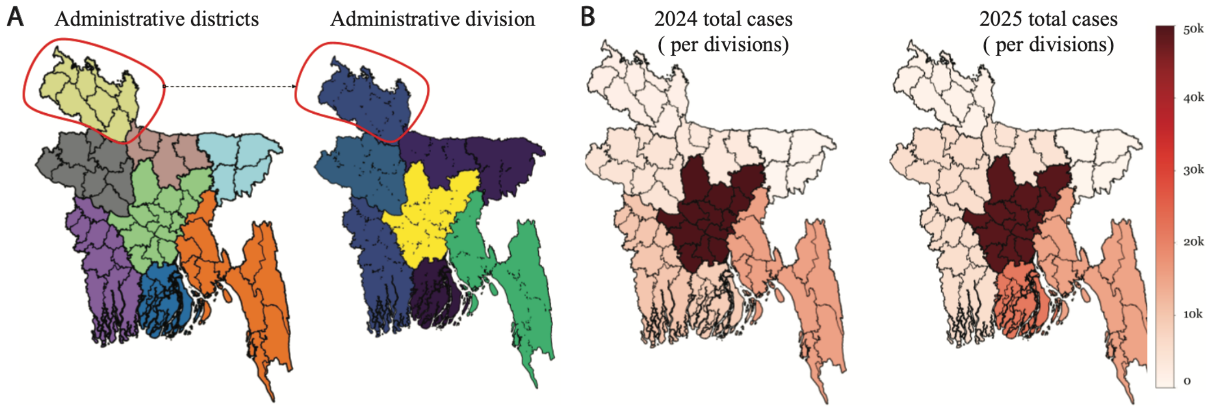

Figure 1. District and division-level aggregation of climate and dengue case data in Bangladesh. Panel (A) shows district-level administrative boundaries (left) and aggregated divisions (right) used for processing climate data (e.g., temperature) and dengue case data, with the northern region highlighted for clarity. Panel (B) presents the total number of reported dengue cases per division for 2024 and 2025. Here, we aggregated district-level data to division-level data by epidemiological week. The color gradient indicates case burden intensity, from low (light colors) to high (dark colors), as shown by the scale bar.

6. We note you have included a table to which you do not refer in the text of your manuscript. Please ensure that you refer to Table 1 in your text; if accepted, production will need this reference to link the reader to the Table.

   Response: We appreciate this comment. A reference to **Table 1** has now been included in the relevant section of the manuscript.

7. Please include captions for your Supporting Information files at the end of your manuscript, and update any in-text citations to match accordingly.

   Response: Captions for the Supporting Information files have been added, and all in-text citations have been updated accordingly.

8. If the reviewer comments include a recommendation to cite specific previously published works, please review and evaluate these publications to determine whether they are relevant and should be cited. There is no requirement to cite these works unless the editor has indicated otherwise.

   Response: Thank you for the guidance. We have reviewed the suggested publications and cited those that are relevant to the scope and content of our manuscript.

Referee# 1 (Remarks to the Author)
Reviewer#1: The authors did a good job, but I have few comments to make:

Overall response to Reviewer#1:

We sincerely thank the reviewer for their positive assessment of our work and for providing constructive and insightful comments. We have carefully addressed each point raised below, and the revisions have substantially improved the clarity, rigor, and presentation of the manuscript. We greatly appreciate the reviewer's encouraging feedback and valuable suggestions, which have helped us refine the manuscript. Detailed responses to each comment are provided below.

1.Add proper reference to the following lines in the Introduction Section:

Response:

We thank the reviewer for this helpful suggestion. We have revised the "Introduction" to include appropriate references supporting each of the indicated statements. Specifically:

a))) The virus persists in the human bloodstream for two to seven days,
[3] [Chawla P, Yadav A, Chawla V. Clinical implications and treatment of dengue. Asian Pacific journal of tropical medicine. 2014 Mar 1;7(3):169-78.]

b))) Globally, dengue imposes a heavy public health burden, with an estimated 400 million infections annually
[8] [Lessa CL, Hodel KV, Goncalves MD, Machado BA. Dengue as a disease threatening global health: a narrative review focusing on Latin America and Brazil. Tropical Medicine and Infectious Disease. 2023 Apr 23;8(5):241.]

c))) Between 1990 and 2021, reported cases increased by 88%,
[9] [Zhang WX, Zhao TY, Wang CC, He Y, Lu HZ, Zhang HT, Wang LM, Zhang M, Li CX, Deng SQ. Assessing the global dengue burden: Incidence, mortality, and disability trends over three decades. PLoS neglected tropical diseases. 2025 Mar 12;19(3):e0012932]

d))) In 2024, cases declined to 101,214 with 575 deaths
[15] [Directorate General of Health Services (Bangladesh). 2024. Daily Dengue Status Report [Accessed 16 June 2024]. https://old.dghs.gov.bd/index.php/bd/home/5200-daily-dengue-status-report]

e))) Importantly, temperature shows the strongest correlation with dengue cases among the climate variables
[28] [Pinto E, Coelho M, Oliver L, Massad E. The influence of climate variables on dengue in Singapore. International journal of environmental health research. 2011 Dec 1;21(6):415-26.]

We hope that these additions strengthen the empirical grounding of the Introduction and improve clarity.

2. In page 6, mention the expression of N_h in the disease free equilibrium (DFE) like N_v.

Response:

We thank the reviewer for pointing out this omission. In the revised manuscript, we have explicitly stated the expression for the total human population at the disease-free equilibrium. At the DFE, all infection-related compartments vanish, and the human population satisfies

$$S_h^* = N_h, I_h^* = T_h^* = R_h^* = 0,$$

with $N_h$remaining constant due to the balance between human recruitment and natural mortality. This clarification has now been added to Section 2.4 to maintain consistency with the presentation of the mosquito population $S_v^* = N_v = \frac{A}{\mu_v}$ at the disease-free equilibrium.

3. Modify the structure of column 6 and column 7 in Table 2. Put the values in column 6 and units in column 7, do not write together.

Response:

We thank the reviewer for this suggestion. Table 2 has been revised so that column 6 now contains the parameter values, and column 7 lists the corresponding units separately. This improves the clarity and readability of the table.

For clarity, Table 2 is shown below.

Table 2: Presenting the simulation's parameter values

Variable	Value	Unit	R_c	Source	Value	Unit	R_c	Source
N_h	17295319	Unitless	3.0456 (based on column 2)	Varies across locations	169828921	Unitless	15.5464 (based on column 6)	[58]
N_v	19080000	Unitless		[57]	19080000	Unitless		[57]
μ_h	2.9586e-04	$Week^{-1}$		Assumed	0.0143<br>0.000275	$Year^{-1}$<br>$Week^{-1}$		[57]
μ_v	0.7019	$Week^{-1}$		Assumed	0.032300<br>0.008	$Month^{-1}$<br>$Week^{-1}$		[48]
γ_h	0.143	$Week^{-1}$		Varies	0.328833<br>0.0822	$Month^{-1}$<br>$Week^{-1}$		[48]
τ_h	0.03940	$Week^{-1}$		Varies	0.53<br>3.71	Day^{-1}<br>$Week^{-1}$		[59]
δ_h	0.10000	$Week^{-1}$		Assumed	0.99977<br>6.99839	Day^{-1}<br>$Week^{-1}$		[60]
θ_h	0.00274	$Week^{-1}$		Assumed	0.575000<br>0.14375	$Month^{-1}$<br>$Week^{-1}$		[48]
β_v	0.85	Unitless		Varies	0.375	Unitless		[61]
β_h	0.85	Unitless		Varies	0.375	Unitless		[61]
b	0.70	$Week^{-1}$		Varies	0.8<br>5.6	Day^{-1}<br>$Week^{-1}$		[61]
ρ	0.1	Unitless		Varies	0.1	Unitless		[62]

4. In column 4 of Table 2, the values of $\boldsymbol{R_c}$ should be based on column 2, not column 3.

Response:

Thank you for pointing this out. We have recalculated the values of the control reproduction number R_c in Column 4 using the parameters listed in Column 2, and Table 2 has been updated accordingly. For clarity, Table 2 is shown below.

Table 2: Presenting the simulation's parameter values

**Variable**	**Value**	**Unit**	$\boldsymbol{R_c}$	**Source**	**Value**	**Unit**	$\boldsymbol{R_c}$	**Source**
N_h	17295319	Unitless	3.0456 (based on column 2)	Varies across locations	169828921	Unitless	15.5464 (based on column 6)	[58]
N_v	19080000	Unitless		[57]	19080000	Unitless		[57]
μ_h	2.9586e-04	$Week^{-1}$		Assumed	0.0143<br>0.000275	$Year^{-1}$<br>$Week^{-1}$		[57]
μ_v	0.7019	$Week^{-1}$		Assumed	0.032300<br>0.008	$Month^{-1}$<br>$Week^{-1}$		[48]
γ_h	0.143	$Week^{-1}$		Varies	0.328833<br>0.0822	$Month^{-1}$<br>$Week^{-1}$		[48]
τ_h	0.03940	$Week^{-1}$		Varies	0.53<br>3.71	Day^{-1}<br>$Week^{-1}$		[59]
δ_h	0.10000	$Week^{-1}$		Assumed	0.99977<br>6.99839	Day^{-1}<br>$Week^{-1}$		[60]
θ_h	0.00274	$Week^{-1}$		Assumed	0.575000<br>0.14375	$Month^{-1}$<br>$Week^{-1}$		[48]
β_v	0.85	Unitless		Varies	0.375	Unitless		[61]
β_h	0.85	Unitless		Varies	0.375	Unitless		[61]
b	0.70	$Week^{-1}$		Varies	0.8<br>5.6	Day^{-1}<br>$Week^{-1}$		[61]
ρ	0.1	Unitless		Varies	0.1	Unitless		[62]

5. In section 2.9, write a clear and rigorous explanation of the sensitivity index. For example, the sensitivity index of $\boldsymbol{R_c}$ with respect to the parameter beta_h is 1, what does it mean?

Response:

Thank you for your comment. We have revised Section 2.9 to provide a clearer and more rigorous explanation of the sensitivity index, including a biological interpretation. For example, when the sensitivity of R_c with respect to a parameter (e.g., β_h) equals 1, it indicates that a 100% change in β_h would result in a proportional 100% change in R_c, highlighting the direct influence of that parameter on disease transmission.

6. What initial conditions were used for each compartment?

Response:
We thank the reviewer for this comment. In the revised manuscript, Section 2.8 has been updated and renamed **"Model parameters and initial conditions."** Following Table 2, we now explicitly state the initial conditions used for each compartment in both simulations and calibration:

- **Simulations:** $S_h(0)$ was set to the actual population size for each division (Table S1); $I_h(0) = 1$, $T_h(0) = 0$, $R_h(0) = 0$; mosquito compartments were initialized with $S_v(0) = N_v - I_v(0)$ and $I_v(0) = 10$.
- **Calibration:** $I_h(0)$ was set based on reported cases in the first epidemiological week, with $\rho = 1/10$. In divisions with zero reported cases (Rajshahi and Rangpur), a single infectious individual was assumed.

These additions make the starting conditions fully explicit and ensure reproducibility of both simulations and calibration.

7. How the parameter values were chosen for different scenarios (low, low-moderate, moderate, high-moderate, very high) in page 17? Were these values just assumed or is there any significance?

Response:
We thank the reviewer for this question. In the revised manuscript (Section 3.3), we clarified that the parameter values for the different infection scenarios were **assumed systematically** to represent a gradient of transmission intensities, ranging from low to very high infection risk. Specifically, the scenarios vary the **mosquito biting rate (b)** and the **transmission probabilities (β_h and β_v)** to explore how changes in these key epidemiological factors affect dengue spread under different levels of treatment coverage and effectiveness.

While the exact numeric values were assumed, they were chosen to be **epidemiologically plausible**: for example, b > 1 reflects higher mosquito biting intensity, which is known to increase transmission [65]. This approach allows us to investigate the **impact of treatment and recovery interventions** across a spectrum of transmission intensities, providing insights into potential outcomes under different risk conditions. The six scenarios are explicitly described in Section 3.3 (Low, Low-Moderate, Moderate, Moderate-High, High, and Very High), with corresponding values of b, β_h, and β_v provided for reproducibility.

Reference: [65] Dahlin KJ, Robert MA, Childs LM (2025) Once bitten, twice shy: A modeling framework for incorporating heterogeneous mosquito biting into transmission models: KJ-M. Dahlin et al. Bulletin of Mathematical Biology 87:163.

8. In the very high infection scenario, b =1.25. Can b be greater than 1? If yes, why?

Response:

We thank the reviewer for this question. We have clarified this in Section 3.3 ("Impact of treatment on infection dynamics across different risk scenarios"), where we explain that b is a rate parameter, not a probability. Values above 1 reflect increased mosquito biting intensity, which is epidemiologically plausible and contributes to higher dengue transmission in the Moderate-High and Very High infection scenarios.

As described in Section 3.3 ("Impact of treatment on infection dynamics across different risk scenarios"), the biting rate b is a rate parameter, not a probability. In the Moderate-High and Very High infection scenarios, b exceeds 1 (e.g., $b = 1.25$) to reflect increased mosquito biting intensity, which is epidemiologically plausible. A value of $b = 1$ corresponds to each mosquito biting once per day, while $b = 1.25$ indicates, on average, 1.25 bites per day per mosquito. Higher biting rates amplify contact between infected vectors and susceptible humans, increasing dengue transmission and contributing to elevated reproduction numbers. These scenarios capture conditions such as high vector density, favorable environmental factors, and increased host availability, as discussed in Section 3.3 and supported by [65].

Reference: [65] Dahlin KJ, Robert MA, Childs LM (2025) Once bitten, twice shy: A modeling framework for incorporating heterogeneous mosquito biting into transmission models: KJ-M. Dahlin et al. Bulletin of Mathematical Biology 87:163.

9. The paper mentions that parameter values were estimated using MATLAB's fmincon. What are the confidence intervals for these estimates, and how were these parameters initialized during the optimization process?

Response:

We thank the reviewer for the question. The parameters were estimated by fitting the model to observed incidence data using MATLAB's fmincon to minimize the sum of squared residuals. The optimization was initialized using plausible epidemiological values based on literature and baseline assumptions.

The parameter values themselves are fixed after optimization; however, we quantified uncertainty in the model predictions using confidence intervals. Specifically, we computed $\pm\sigma$ intervals around the fitted model output to generate 95%, 90%, 80%, and 50% confidence intervals, as shown in Fig. 11. These intervals reflect the variability in the model predictions given the estimated parameters and the residual error, rather than uncertainty in the parameter estimates themselves.

This approach allows readers to assess the reliability of the model simulations while acknowledging that the fitted parameters are fixed.

10. In page 10, the paper mentions Routh–Hurwitz criterion, but neither stated it nor gave any references.

Response:

Thank you for your comment. We have now explicitly stated the Routh–Hurwitz criterion in the manuscript and included an appropriate reference to support its use:

[56] Morris J (1996) The routh and routh-hurwitz stability criteria: Their derivation by a novel method using comparatively elementary algebra. Aircraft Engineering and Aerospace Technology 34:25-7.

Referee # 2 (Remarks to the Author)

Overall response to Reviewer#2:

We sincerely thank Reviewer #2 for your thoughtful and constructive comments on our manuscript. The suggestions provided were extremely helpful in improving the clarity, rigor, and presentation of our work. We have carefully considered each point and revised the manuscript accordingly. Below, we provide a detailed point-by-point response to all comments. We greatly appreciate the reviewer's insightful questions and recommendations, which have helped us refine the manuscript.

Reviewer #2: Minor Comments:
1.The manuscript contains several spelling inconsistencies (e.g. suppohomorting) that should be carefully reviewed and corrected throughout.

Response:

Thank you for your comment. The manuscript has been thoroughly proofread, and all spelling inconsistencies and typographical errors, including the example provided, have been corrected throughout the text.

2. The reference numbering does not appear to follow a consistent order throughout the manuscript. Please review and revise the placement of references to ensure proper sequencing.

Response:

Thank you for your comment. The reference numbering has been carefully reviewed and revised to ensure consistent and proper sequencing throughout the manuscript.

3. A careful check of the reference list is recommended, as certain references seem to appear repeatedly.

Response:

Thank you for your comment. The reference list has been carefully reviewed and revised to remove any repeated or duplicate entries.

4. The parameter τ_h is inconsistently described as a specified time interval, a disease progression rate, and a treatment rate in different sections. Clarification and revision are requested to ensure consistency.

Response:

Thank you for your comment. The parameter τ_h has been consistently defined throughout the manuscript, clarifying its role and ensuring uniform interpretation in all sections.

5. Please check the formatting of columns 6 and 7 of table 2, as they appear to be merged.

Response:

Thank you for your comment. The formatting of columns 6 and 7 in Table 2 has been corrected, and the columns are now properly separated for clarity. For clarity, Table 2 is shown below.

Table 2: Presenting the simulation's parameter values

<table>
<tr><th>Variable</th><th>Value</th><th>Unit</th><th>R_c</th><th>Source</th><th>Value</th><th>Unit</th><th>R_c</th><th>Source</th></tr>
<tr><td>N_h</td><td>17295319</td><td>Unitless</td><td rowspan="5">3.0456 (based on column 2)</td><td>Varies across locations</td><td>169828921</td><td>Unitless</td><td rowspan="5">15.5464 (based on column 6)</td><td>[58]</td></tr>
<tr><td>N_v</td><td>19080000</td><td>Unitless</td><td>[57]</td><td>19080000</td><td>Unitless</td><td>[57]</td></tr>
<tr><td rowspan="2">μ_h</td><td rowspan="2">2.9586e-04</td><td rowspan="2">$Week^{-1}$</td><td rowspan="2">Assumed</td><td>0.0143</td><td>$Year^{-1}$</td><td rowspan="2">[57]</td></tr>
<tr><td>0.000275</td><td>$Week^{-1}$</td></tr>
<tr><td>μ_v</td><td>0.7019</td><td>$Week^{-1}$</td><td>Assumed</td><td>0.032300</td><td>$Month^{-1}$</td><td>[48]</td></tr>
</table>

					0.008	$Week^{-1}$		
γ_h	0.143	$Week^{-1}$		Varies	0.328833	$Month^{-1}$		[48]
					0.0822	$Week^{-1}$		
τ_h	0.03940	$Week^{-1}$		Varies	0.53	Day^{-1}		[59]
					3.71	$Week^{-1}$		
δ_h	0.10000	$Week^{-1}$		Assumed	0.99977	Day^{-1}		[60]
					6.99839	$Week^{-1}$		
θ_h	0.00274	$Week^{-1}$		Assumed	0.575000	$Month^{-1}$		[48]
					0.14375	$Week^{-1}$		
β_v	0.85	Unitless		Varies	0.375	Unitless		[61]
β_h	0.85	Unitless		Varies	0.375	Unitless		[61]
b	0.70	$Week^{-1}$		Varies	0.8	Day^{-1}		[61]
					5.6	$Week^{-1}$		
ρ	0.1	Unitless		Varies	0.1	Unitless		[62]

6. Please carefully check the x-tick labels in Fig. 2, Panel B, as they may require correction.

Response:

Thank you for your comment. The x-axis labels in Fig. 2, Panel B, have been carefully reviewed and corrected. The axis now explicitly indicates time in weeks, with clear demarcation of the corresponding weeks across different years.

Figure 2 has been updated and is shown below for your reference.

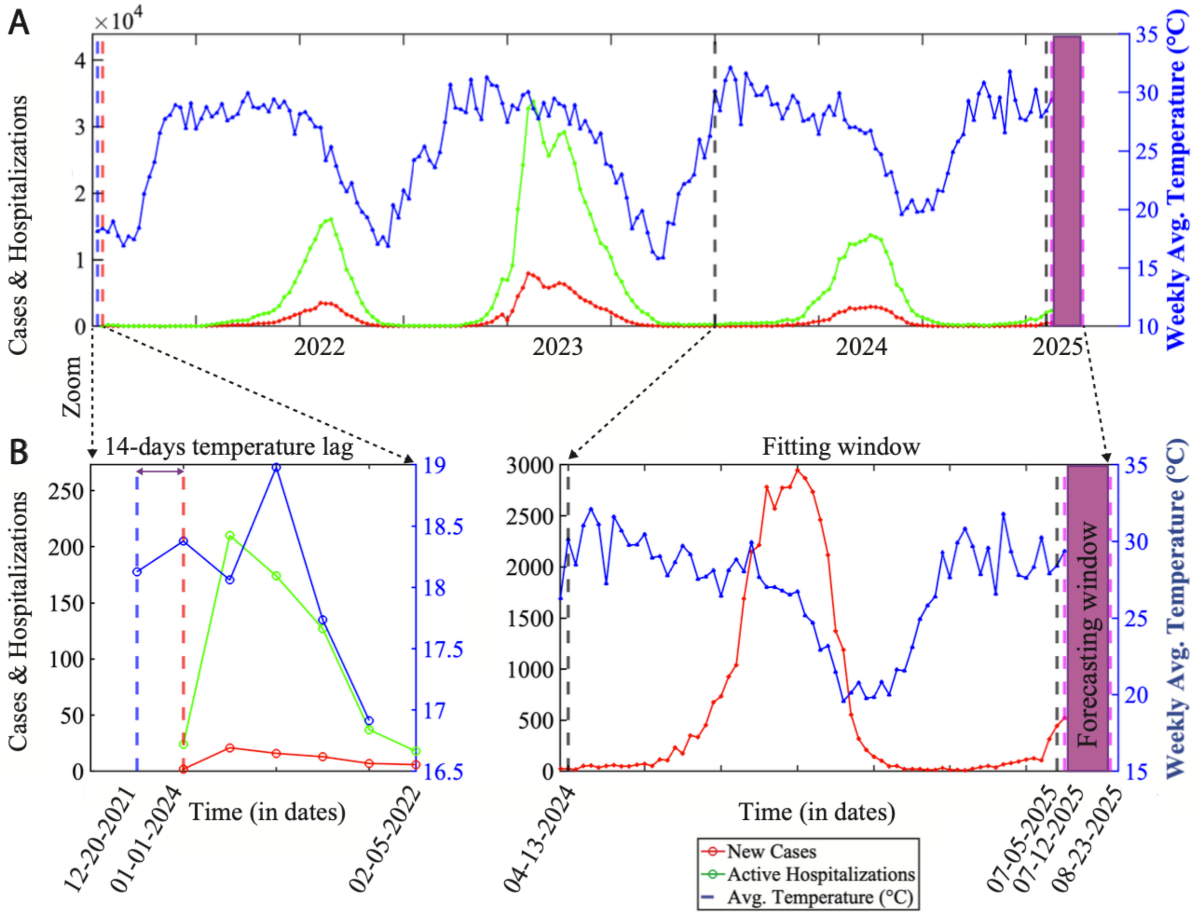

Figure 2. Weekly dengue cases, hospitalizations, and average temperature in the Dhaka Metropolitan Area (2021–2025). Panel A shows the time series of reported dengue cases in the Dhaka metropolitan area from January 1, 2022, to August 23, 2025. The lower-left part of Panel B provides a zoomed view, illustrating the fit of reported dengue cases using the past 14-day averaged temperature ($°C$) to define the mosquito biting rate function $b(T)$. The right part of panel B shows zoomed view of the Dhaka metropolitan dataset into two phases: a fitting phase, spanning April 13, 2024, to July 05, 2025 (EW 15, 2024 to EW 27, 2025), and a forecasting phase, covering July 12, 2025, to August 23, 2025 (EW 28, 2025 to EW 34, 2025).

7. Please carefully revise the theorem (Theorem 3) statement to ensure consistency between the stability condition and the biological interpretation.

Response:

Thank you for your comment. The statement of Theorem 3 has been carefully revised to ensure that the mathematical stability condition is fully consistent with its biological interpretation.

Major Comments:

1.Given that Fig. 8 depicts scenarios for human infections (I_h). Clarification is requested on how conclusions regarding infection dynamics in both human and mosquito populations were drawn, and what analyses were conducted to assess the role of mosquito control under epidemic conditions.

Response:

Thank you for your comment. Human and mosquito individuals were taken from the host–vector model and evaluated using scenario-based simulations that reduced mosquito infection levels. These simulations allowed us to assess the role of mosquito control under epidemic conditions and to draw conclusions about infection dynamics in both human and mosquito populations. **Figure 8 has been updated accordingly to reflect these analyses as shown below.**

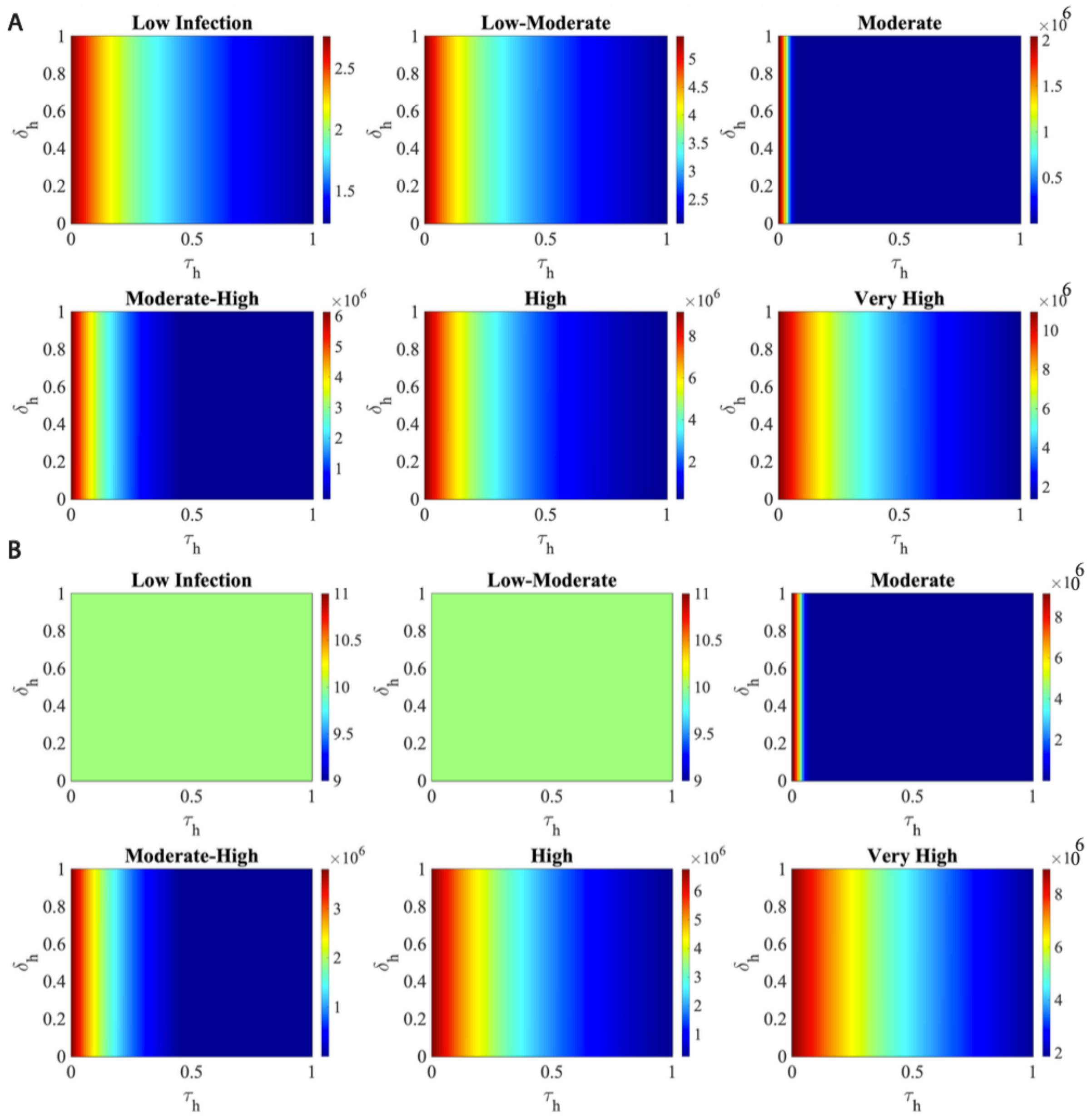

Figure 8. Peak infected dynamics for human and vector populations across six infection-risk scenarios with varying treatment and recovery rates. The figure presents a multidimensional sensitivity analysis of parameters $\boldsymbol{\tau_h}$ and $\boldsymbol{\delta_h}$ across six increasing infection severity levels. In **Panel A**, the horizontal gradient indicates high sensitivity to $\boldsymbol{\tau_h}$, with minimal response to variations in $\boldsymbol{\tau_h}$. As $\boldsymbol{\tau_h}$ approaches zero, the outcome peaks sharply-transitioning into the red spectrum-while larger values lead to rapid decline toward baseline. Infection severity markedly boots the outcome, increasing from approximately 2.5 to over $10 * 10^6$ from "Low" to "Very High" severity. **Panel B** exhibits a distinct regime shift in vector population: early infection stages ("Low" and "Low-Moderate") show uniform parameter insensitivity (green heatmaps), whereas beyond the "Moderate" threshold, sensitivity to $\boldsymbol{\tau_h}$ re-emerges, mirroring Panel A's vertical line. This suggests a threshold effect whereby $\boldsymbol{\tau_h}$ influences system dynamics only past a critical severity level. Throughout, the negligible vertical color variation confirms $\boldsymbol{\tau_h}$ as the dominant parameter, with $\boldsymbol{\delta_h}$ exerting minimal impact within this parameter space.

2. Could you please clarify how the analysis presented in Fig. 9 supports conclusions regarding infection dynamics in the mosquito population? While treatment may indirectly reduce mosquito infections by lowering human infectiousness, it would be helpful to explain how such conclusions were drawn in the absence of explicit vector control interventions or mosquito-targeted measures in the model.

Response:

Thank you for your comment. The change in mosquito infection dynamics emerges indirectly through decreased human-to-mosquito transmission due to treatment, coupled with temperature-dependent biting, rather than through explicit vector control measures. Figure 9 has been revised to clarify how these dynamics in the mosquito population are influenced by interventions targeting humans.

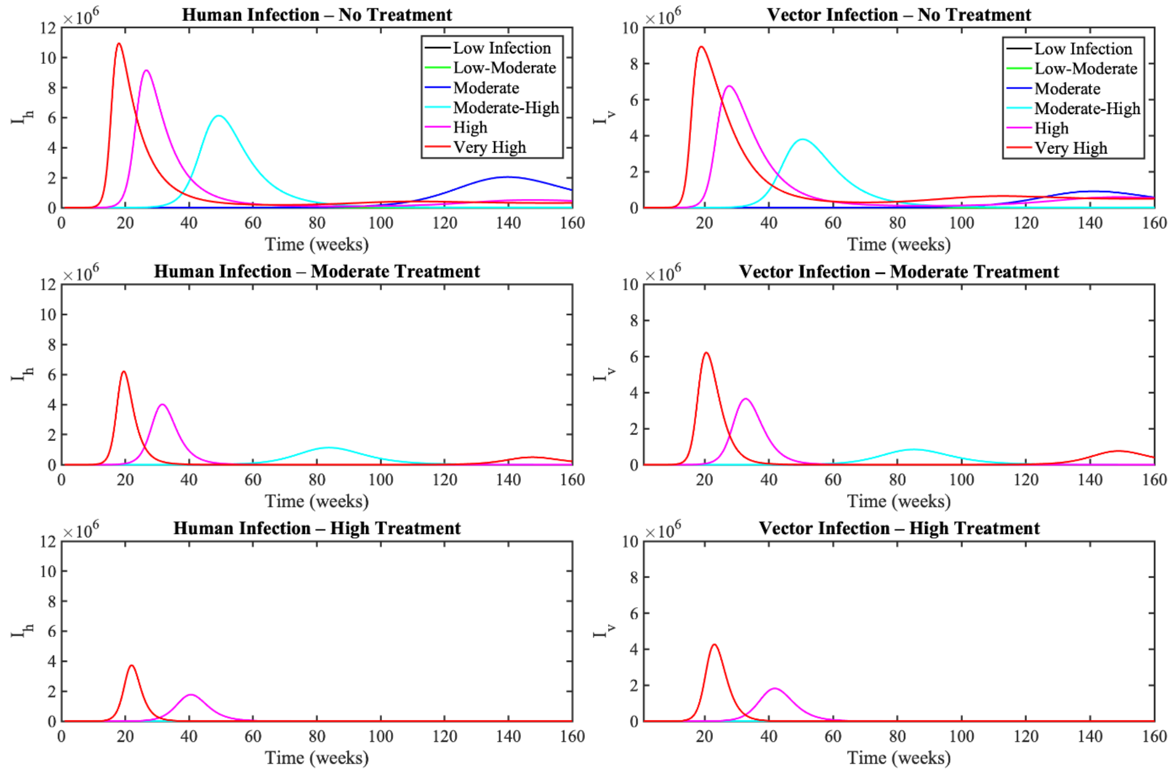

Figure 9. Impact of varying treatment levels on infection dynamics in human and vector populations across six risk scenarios. The simulations examine how varying levels of treatment impact the spread of infection in both humans and mosquitoes across six scenarios, ranging from low to very high infection risk. Infection levels (I_h *and* I_v) escalate rapidly, with the red (Very High) and magenta (High) for the first 40 weeks. We examined three treatment regimens: *no treatment* (treatment rate, $\tau_h = 0$ and recovery rate, $\delta_h = 0$) *moderate treatment* ($\tau_h = 0.25, \delta_h = 0.5$) and *strong treatment* ($\tau_h = 0.5, \delta_h = 0.9$). Without treatment, infections rapidly increase and reach high peaks, especially in the higher-risk scenarios. Introducing moderate and high treatments significantly reduces the number of infections and shortens outbreak duration, with the greatest impact observed in lower-risk scenarios. Under intense treatment, infection peaks are much smaller, and outbreaks progress more slowly across all scenarios, demonstrating the potential to control even very high transmission levels. These results highlight that both the extent of treatment coverage and its effectiveness play critical roles in managing disease spread in human and mosquito populations.

3. The temperature-dependent biting rate function b(T) is directly adopted from reference [43]. Could you please clarify the rationale for applying this formulation in the Bangladesh context? In particular, clarification is requested regarding the suitability of the adopted formulation for Bangladesh-specific conditions and whether any validation or sensitivity analyses were performed.

Response:

Thank you for your comment. To ensure the model reflects Bangladesh-specific conditions, we used real temperature data aggregated at the district level for each division. As shown in Figure S2 (Appendix), temperature consistently exhibits the strongest positive correlation with weekly dengue cases across all regions, compared with other climatic variables such as relative humidity, dew point, and rainfall. This supports the application of the temperature-dependent biting rate function $b(T)$in capturing the mechanistic influence of temperature on mosquito biting, development, and extrinsic incubation period. By using actual weekly average temperatures from the study locations to calculate $b(T)$, the model accounts for local temperature variability and provides a Bangladesh-specific measure of transmission dynamics. Supplementary analyses also show that $b(T)$ correlates more strongly with incidence than raw temperature values, justifying its use in the model [13, 66].

4.Figure 5, Panel A is described as using the baseline parameter values listed in Table 2; however, Table 2 reports a basic reproduction number $R_c=3.0456$ for these parameter values, whereas Figure 5 indicates $R_c<1$. Could you please clarify this apparent discrepancy and explain how the condition $R_c<1$ was obtained in Figure 5?

Response:

Thank you again for your comment. We have clarified the figure caption and corresponding text. Panel A of Figure 5 uses modified transmission parameter values chosen to achieve $R_c < 1$ for illustrative purposes, while Panel B corresponds to the baseline parameter values reported in Table 2, where $R_c = 3.0456$. This distinction has now been explicitly stated to resolve the apparent discrepancy.

Figure 5 has been updated and is shown below for your reference.

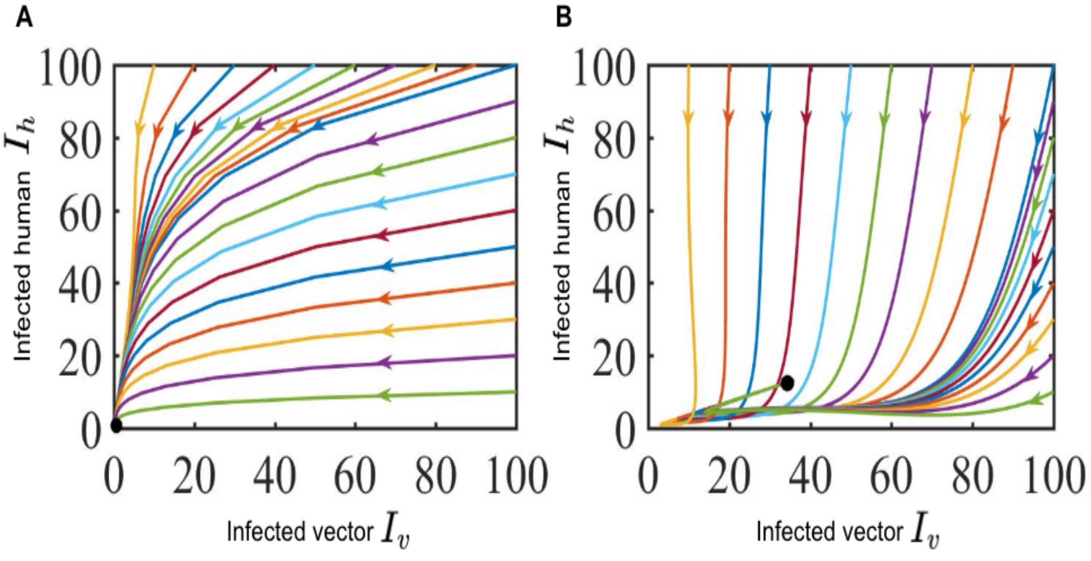

Figure 5. Impact of disease dynamics for $R_c^{NG} < 1$ and $R_c^{NG} > 1$. This figure shows the disease dying out($R_c^{NG} < 1$) and the endemic state ($R_c^{NG} > 1$). Panel A used the baseline parameter values given in Table 2, while the panel B used the parameter values: $\mu_h = 0.01$; $\gamma_h = 0.05$; $\tau_h = 0.07$; $\delta_h = 0.05$; $\mu_v = 0.025$;$\theta_h = 0.01$; $N_h = 15295319$; $N_v = 20080000$; $c = 0.07$; $\beta_h = 0.755$; $\beta_v = 0.855$.

5. The figure description (Fig. 6) appears to be inconsistent with the content shown in the figure. Clarification or revision would be appreciated to ensure consistency between the figure and its description.

Response:

Thank you for your comment. The description of Figure 6 has been carefully revised to ensure it is fully consistent with the content displayed in the figure.

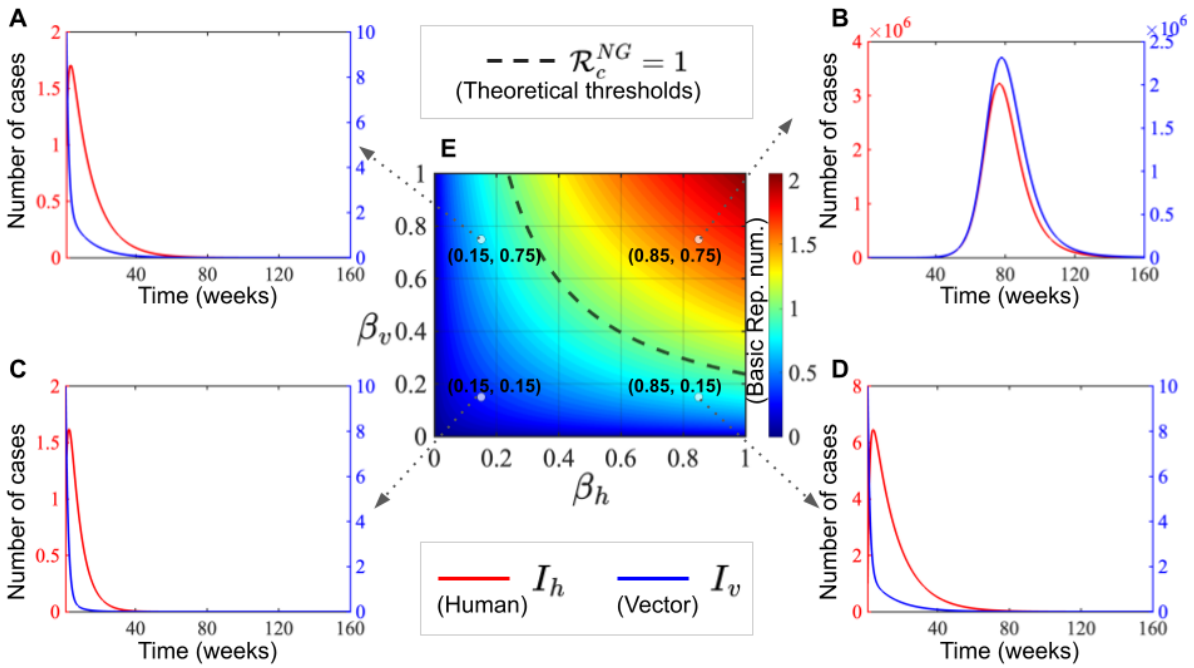

Figure 6. Transmission dynamics of dengue modeled through human and vector infections over time (weeks) and parameter space analysis. This figure illustrates the colour map of the theoretical basic reproduction number R_c^{NG} as a function of β_h and β_v, both ranging from 0 to 1, along with plots of the theoretical threshold at $R_0 = 1$. The other parameters are fixed as follows: $\mu_h = 2.9586e-04$, $\mu_v = 0.7019$, $\tau_h = 0.03940$, $\gamma_h = 0.143$, $b = 0.70$ and population sizes $N_h = 17295319$, $N_v = 19080000$, are fixed. The simulation highlights the disease dynamics in humans (red solid line) and vectors (blue solid line), demonstrating the potential for both short-term and long-term outbreaks. Most importantly, the duration and intensity of outbreaks depend

on the values of β_h and β_v within the simulation. The simulated parameters and initial values are same to those listed in Table 2. Panels (A), (C), and (D) show the temporal evolution of infected humans (red curves) and vectors (blue curves) under different transmission parameters β_h (human) and β_v (vector). Panel (B) depicts the case dynamics when transmission rates produce epidemic peaks. Panel (E) presents a heatmap of the basic reproduction number R_c^{NG} as a function of β_h (human) and β_v (vector), with the theoretical threshold $R_c^{NG} = 1$ indicated by the dashed line. Specific parameter combinations are annotated on the heatmap, illustrating their relative positions in the transmission landscape.

6. While sensitivity analyses of epidemiological parameters in human and vector classes are presented, the inclusion of an LHS–PRCC approach could further account for parameter uncertainty.

Response:

Thank you for your comment. We have now included a Latin Hypercube Sampling–Partial Rank Correlation Coefficient (LHS–PRCC) analysis to better account for parameter uncertainty and assess the robustness of the model outcomes. The results are presented in **Fig. 4** and reproduced below, illustrating the relative influence of each parameter on both human and vector classes. This provides a clearer understanding of parameter sensitivity under uncertainty.

Figure 4 has been updated and is shown below for your reference.

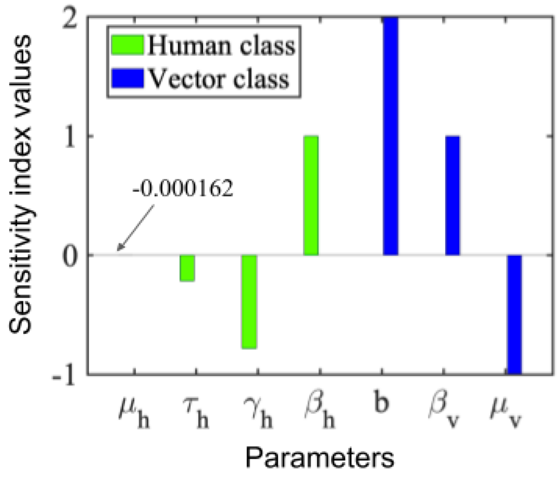

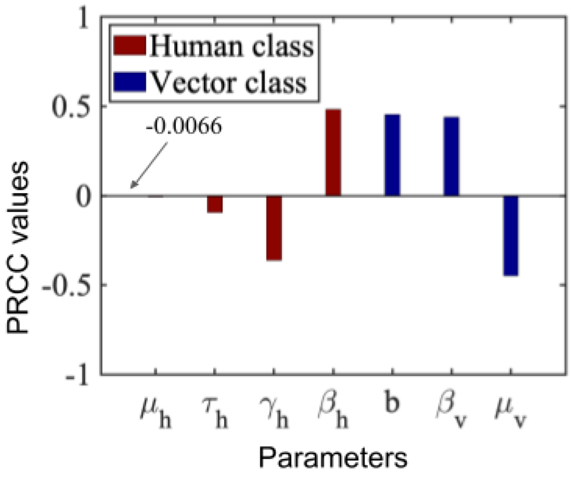

Figure 4. Sensitivity index (left) and Partial Rank Correlation Coefficient (right) for model parameters in human and vector classes, illustrating the relative influence of each parameter on model outcomes. The **left panel** in Fig. 4 shows the normalized sensitivity indices for the model parameters. Positive values indicate parameters that increase R_c when they increase, while negative values indicate parameters that decrease R_c. For the human class, the biting rate b and transmission rate β_h have the largest positive impact, whereas the natural recovery rate γ_h has a negative effect. For the vector class, the transmission rate β_v positively influences outcomes, while the mosquito birth and mortality rate μ_v negatively affects them. For example, a 100% increase in β_h would result in an approximately 100% increase in R_c.

The **right panel** shows the PRCC values of model parameters with respect to model output. Positive PRCC values indicate a favorable association, while negative values indicate an adverse effect. Parameters b, β_h, and β_v are strongly positively correlated with the output, whereas γ_h and μ_v are strongly negatively correlated. Parameters μ_h and τ_h show minimal or moderate influence. Overall, both sensitivity index and PRCC analyses consistently indicate that $b, \beta_h, \beta_v, \gamma_h$, and μ_v are the most influential parameters, while μ_h has comparatively little impact on model outcomes.

7. Please consider placing figures within their corresponding sections in the main manuscript and integrating the correlation figures from the supporting file where appropriate.

Response:

Thank you for your comment. The figures have been repositioned within their corresponding sections in the main manuscript, and the correlation figures from the supporting file have been integrated where appropriate.

8. The manuscript would benefit from engagement with additional literature on dengue transmission, treatment-based intervention modeling, and uncertainty quantification, as well as broader infectious disease modelling studies on host–vector dynamics, threshold analysis, and control strategies.

a) Cost-effectiveness of dengue control strategies in Bangladesh: An optimal control and ACER-ICER analysis. https://doi.org/10.1016/j.actatropica.2025.107587
b) Optimal control analysis of COVID-19 transmission model with physical distance and treatment. http://dx.doi.org/10.26855/abr.2022.12.001.
c) Analysis of a Data-Driven Vector-Borne Dengue Transmission Model for a Tropical Environment in Bangladesh. https://doi.org/10.1155/2024/2959770
d) Stochastic analysis of Mpox epidemiology with vaccination strategies and environmental persistence. https://doi.org/10.1038/s41598-025-28135-x

Response:

Thank you for sharing these recent and insightful papers. We have reviewed them carefully and incorporated relevant findings into our manuscript, improving the discussion on dengue transmission, treatment-based interventions, uncertainty quantification, and host–vector dynamics. The references have been appropriately cited in the revised manuscript to strengthen our analysis and contextualize our results within the broader literature.

Recommendation

I recommend acceptance of the manuscript after the authors have adequately addressed the major and minor issues outlined above. With these revisions, the manuscript will be substantially strengthened and better positioned to make a meaningful contribution to the field.

Response:

We thank the reviewer for the positive assessment and recommendation. We have carefully addressed all major and minor comments raised and revised the manuscript accordingly. These revisions have strengthened the clarity, rigor, and overall contribution of the work.
